# Machine learning cross-platform proteomic imputation enables protein quality scoring and replication of epidemiological associations

**DOI:** 10.64898/2026.05.05.723059

**Published:** 2026-05-09

**Authors:** Linke Li, Ahmed Alaa, Youxin Tan, Ilker Demirel, Samuel Friedman, Qiayi Zha, Russell Tracy, Kent D. Taylor, Bing Yu, Christie M. Ballantyne, Rajat Deo, Ruth Dubin, Michael Y. Tsai, Gina M. Peloso, Jennifer Brody, Tom Austin, Bruce M. Psaty, Jayna Nicholas, Laura M. Raffield, Usman Tahir, Josef Coresh, Whitney Hornsby, Andrew Chan, Stephen S. Rich, Jerome I. Rotter, Peter Ganz, Robert Gerszten, Anthony Philippakis, Pradeep Natarajan, Zhi Yu

**Affiliations:** 1. Clinical and Translational Epidemiology Unit, Massachusetts General Hospital, Boston, MA; 2. Program in Medical and Population Genetics and the Cardiovascular Disease Initiative, Broad Institute of Harvard and MIT, Cambridge, MA; 3. Department of Electrical Engineering and Computer Science, University of California, Berkeley, Berkeley, CA; 4. University of California, San Francisco, San Francisco, CA; 5. Department of Biostatistics, Harvard T.H. Chan School of Public Health, Boston, MA;; 6. Massachusetts Institute of Technology, Cambridge, MA; 7. ML4H, Broad Institute of Harvard and MIT, Cambridge, MA; 8. Department of Pathology and Laboratory Medicine, Larner College of Medicine, University of Vermont, Burlington, VT; 9. The Institute for Translational Genomics and Population Sciences, Department of Pediatrics, The Lundquist Institute for Biomedical Innovation at Harbor-UCLA Medical Center, Torrance, CA; 10. Department of Epidemiology, Human Genetics and Environmental Sciences, School of Public Health, University of Texas Health Science Center at Houston, Houston, TX; 11. Department of Medicine, Baylor College of Medicine, Houston, TX; 12. Division of Cardiovascular Medicine, Perelman School of Medicine at the University of Pennsylvania, Philadelphia, PA; 13. Department of Medicine, University of Texas Southwestern Medical Center, Dallas, TX; 14. Department of Laboratory Medicine & Pathology, University of Minnesota Medical School, Minneapolis, MN; 15. Department of Biostatistics, Boston University School of Public Health, Boston, MA; 16. Cardiovascular Health Research Unit, Department of Medicine & Deparment of Epidemiology, University of Washington, Seattle, WA; 17. Department of Genetics, University of North Carolina at Chapel Hill, Chapel Hill, NC; 18. Division of Cardiovascular Medicine, Beth Israel Deaconess Medical Center, Boston, MA; 19. Department of Medicine, NYU Grossman School of Medicine, New York, NY, USA; 20. Heart and Vascular Institute, Mass General Brigham, Boston, MA; 21. Department of Medicine, Harvard Medical School, Boston, MA; 22. Department of Genome Sciences, University of Virginia, Charlottesville, VA; 23. Division of Cardiology, Zuckerberg San Francisco General Hospital and Department of Medicine, University of California San Francisco, San Francisco, CA; 24. Google Ventures, Cambridge, MA

## Abstract

High-throughput affinity-based proteomics has advanced biomedical research, yet fundamental, persistent discordance between mainstream platforms (SomaScan and Olink) routinely undermines the replication of findings. This platform-driven non-replication complicates downstream biological validation and biomarker prioritization. Here, we develop a machine learning-based framework for cross-platform protein value imputation to resolve this translational bottleneck. Using paired proteomic data measured by both SomaScan and Olink from 5,325 participants of the Multi-Ethnic Study of Atherosclerosis, we developed models to impute cross-platform measurements and applied them to two independent and demographically distinct cohorts (Cardiovascular Health Study [N=3,171] and UK Biobank [UKB; N=41,405]) for external validation. Our bi-directional model 1) established an imputation performance-based protein fidelity index, validated against gold-standard measurements from Atherosclerosis Risk in Communities study (N=101) and Nurses’ Health Study (N=54), 2) enabled imputation of platform-exclusive protein measurements, and 3) facilitated calibration of overlapping proteins. We demonstrate the utility of this framework through three applications: 1) fidelity-informed analyses enhanced the replication of biomarker discovery, 2) recovery of SomaScan signals that were previously inaccessible in UKB’s original Olink measurements, and 3) improved replication performance for overlapping proteins. Our study offers a translational roadmap that allows researchers to achieve reliable epidemiological replication, target specific assays for future optimization, and prioritize biological signal over platform noise.

## INTRODUCTION

The identification and quantification of circulating plasma proteins have become central to advancing biomarker discovery, particularly in large-scale epidemiological studies^1–3^. High-throughput affinity-based proteomics platforms, which measure thousands of circulating proteins, have greatly accelerated biomedical research in the identification of biomarkers and potential drug targets^3,4^. Two widely adopted high-throughput measuring services are SomaScan (SomaLogic, Inc) and Olink (Thermo Fisher Scientific, Inc).SomaScan utilizes a DNA-based approach of slow off-rate modified aptamers (SOMAmers)^5^, while Olink platform adopts an antibody-based approach known as proximity extension assay (PEA)^6^.

This rapid expansion in proteome measurements has advanced population and medical research at an unprecedented scale, but it has also introduced significant challenges. While affinity-based platforms have excelled beyond traditional methods like enzyme-linked immunosorbent assay (ELISA) in scale and efficiency, this approach comes with an inherent trad e-off. Their reliance on secondary probe-binding signals, rather than direct molecular detection of peptide mass, makes them highly susceptible to off-target interactions driven by protein-altering variants, platform-specific artifacts, and complex structural conformations^7,8^. Consequently, correlations between measurements are frequently modest to low, not only between SomaScan and Olink, but also when compared against gold-standard measures. This leads to the non-replication of associations in studies using different platforms^4,9–11^. Another contributor to this lack of replication is the limited number of shared proteins between platforms and panels^12^. For instance, between SomaScan v4.0 (~5000 proteins) and Olink Explore 3072 (~3000 proteins), only ~1,800 appear on both panels. Naturally, the thousands of proteins unique to each platform are inaccessible for orthogonal validation. As a result, researchers are often left unable to distinguish whether the non-replication arose from false discovery or simply discrepancies between the measuring platforms.

**Figure 1.**
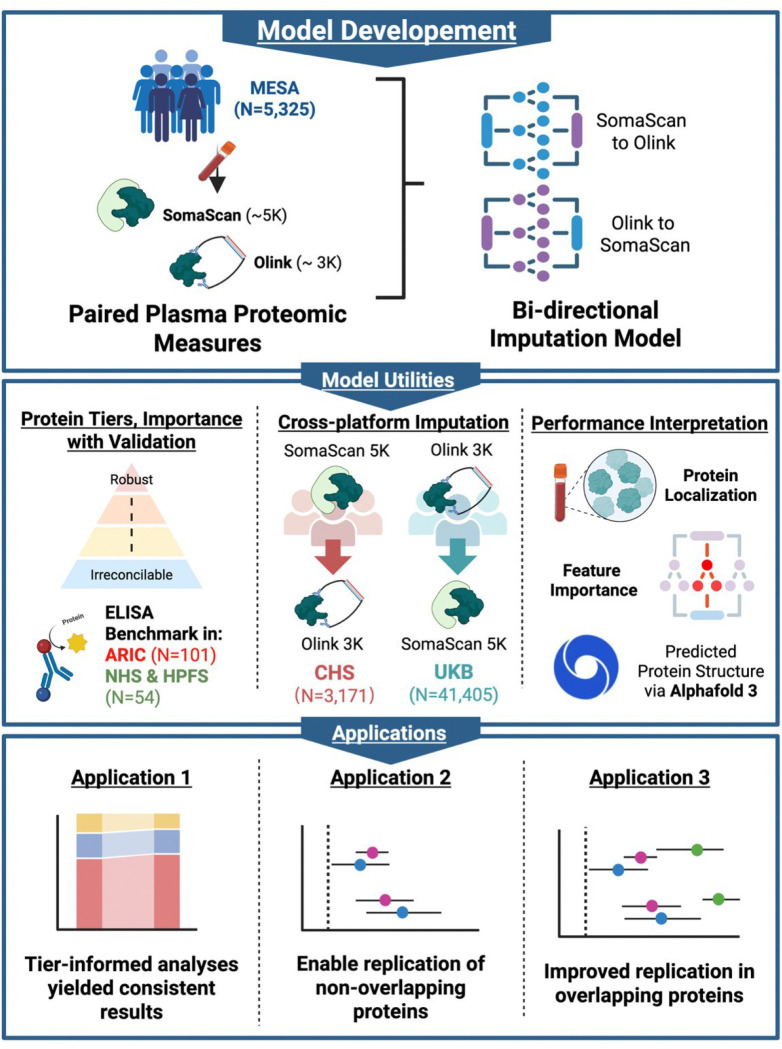
Scheme of Study Design. We developed bi-directional imputation models using paired SomaScan and Olink measurements from MESA. The models would impute the measurements from another platform in single-platform cohorts using measured values (CHS, UKB). We also proposed a tier system for protein measurement qualities and model recoverability and supported the various protein performances using empirical validations such as protein class (secreted / plasma, membrane, intracellular), ELISA, feature importance, and predicted protein structure by Alphafold 3. The validity of the imputed values and the tier system was further tested in 3 real-world applications: 1) tier-informed analyses yielded highly replicable and consistent results, 2) imputation enables validation of non-overlapping proteins in single-platform cohorts, and 3) imputed values improved replication of signals compared to using the naive measures. MESA: Multi-Ethnic Study of Atherosclerosis; ELISA: enzyme-linked immunosorbent assay; ARIC: Atherosclerosis Risk in Communities Study; NHS & HPFS: Nurses’ Health Study and Health Professionals Follow-Up Study; CHS: Cardiovascular Health Study; UKB: UK Biobank. This figure was created with BioRender.com

This necessitates a systematic and translational framework to evaluate protein measurement reliability and bridge the gap in coverage between technologies. To address these challenges, we leveraged machine learning approaches and paired proteomic measurements of the same samples from the Multi-ethnic Study of Atherosclerosis (MESA)^13^. By training on these concurrent proteomics data, our models captured the relationships between the two technologies in a data-driven fashion. This framework allowed us to 1) conduct cross-platform imputation and 2) establish a protein tiered reliability system based on cross-platform fidelity that provides a roadmap for reproducible translational research. To validate the translational utility of our frame work, we successfully improved replication of published epidemiological associations across independent cohorts with disjoint proteomic and demographic profiles: UK Biobank (UKB)^3,14,15^ (Olink-only) and Cardiovascular Health Study (CHS)^16,17^ (SomaScan-only), effectively addressing the reproducibility bottleneck induced by platform-driven discordance (Figure 1).

## RESULTS

### Study Population and Protein Processing

To establish a robust framework for cross-platform proteomic imputation and quality scoring, we curated and harmonized data from three large-scale cohorts: the Multi-Ethnic Study of Atherosclerosis (MESA), the UK Biobank (UKB), and the Cardiovascular Health Study (CHS) (Supplementary Table 1, Supplementary Figure 1). The MESA cohort is our dataset for model development, providing a resource of 5,325 participants with concurrent proteomic measurements from both SomaScan (v4.1, ~7000 proteins) and Olink (Explore 3072, ~3000 proteins) platforms. We randomly divided MESA into a training set (N=3,993) for model development and a held-out internal testing set (N=1,332). After quality control, UKB (N=41,405; Olink Explore 3072, ~ 3000 proteins) and CHS (N=3,171; SomaScan v4.0, ~5000 proteins) were used as external validation sets (Supplementary Figure 1).

Supplementary Table 1 displays the characteristics of the three demographically diverse cohorts. The three study populations span different life stages, with mean ages of 57.3 years (standard deviation [SD]=8.19) in UKB and 62.2 years (SD: 10.3) in MESA, compared to a significantly older CHS cohort (mean age: 74.4 [4.9]). The majority of UKB and CHS participants (UKB: 93.1%, CHS: 83.5%) are white, and MESA participants have higher diversity in self-reported race and ethnicity, including representation from Black (25.3%) and Hispanic/Latino (23.0%) participants.

We standardized protein measurements across the three cohorts to define the platform-specific and shared feature spaces (Supplementary Figure 1). SomaScan aptamers were harmonized to the v4.0 panel using SomaDataIO^18^ lift-over to ensure consistency between MESA and CHS, resulting in a final set of 4,973 SomaScan proteins. For Olink, 2,871 proteins remained after filtering for missingness. The intersection of these two platforms yielded 1,737 overlapping proteins (35% of SomaScan proteins and 60% of Olink proteins).

### Cross-platform Correlation, Predictive Modeling, Performance by Protein Localization

Consistent with previous reports^4,10–12,19–21^, baseline concordance between raw SomaScan and Olink measurements was limited. Among the 1,737 overlapping proteins in MESA, the mean Pearson correlation (r) was 0.36, with the majority of proteins (N=988, 57%) exhibiting below moderate correlation (r<0.4) and only 334 (19%) proteins showing strong agreement (r>0.7). This baseline discordance varied across the proteome, ranging from null (r=0.0001 for CRISP3 [cysteine-rich secretory protein 3]) to highly correlated (r=0.95 for SIGLEC5 [sialic acid-binding Ig-like lectin 5]).

To bridge this gap, we used histogram-based gradient boosting (HGB) regressors trained on paired MESA data to develop a bi-directional imputation framework. This approach enabled the bi-directional imputation of measurements of Olink using SomaScan (SO) and of SomaScan using Olink (OS) in single-platform cohorts such as UKB and CHS, thereby avoiding the forced integration of potential mismeasured values.

**Figure 2.**
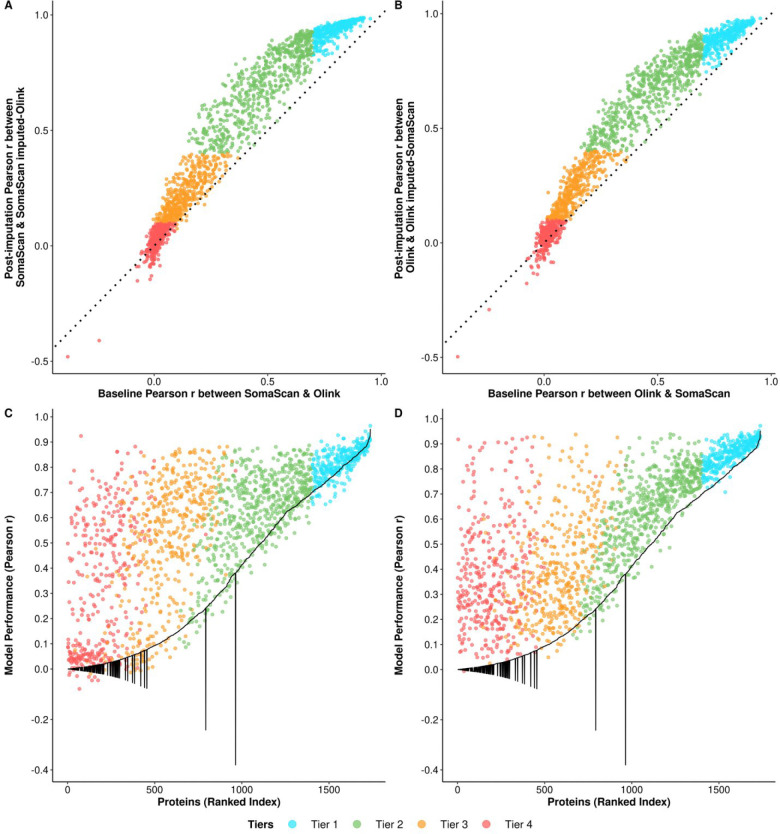
**Comparison of baseline and post-imputation protein correlations in the MESA cohort of 1,737 overlapping proteins. Scatter plots A. SomaScan to Olink direction, B. Olink to SomaScan direction -** Scatter plots comparing baseline correlation (Pearson correlation of paired measured SomaScan and measured Olink values) and post-imputation correlation (Pearson correlation between measured SomaScan and SomaScan imputed-Olink in SomaScan to Olink direction, and between measured Olink and Olink-imputed SomaScan in Olink to SomaScan direction). Each point represents an individual protein, colored by its tier membership. The black dotted identity line x=y) indicates no change in correlation; points above this line represent proteins showing improved correlation post imputation, and vice versa. **Scatter plot C. SomaScan to Olink direction, D. Olink to SomaScan direction -** Model predictive performance in relation to baseline correlation. The predictive power of the model is defined as the Pearson correlation between model-imputed value and measured value in the MESA held-out test set. Proteins are ranked by their baseline Pearson correlation squared along the x-axis to illustrate the magnitude of baseline correlation, including negative correlations. The black line represents baseline correlation. Each point, colored by proteins’ respective tier, denotes the model performance of that protein in relation to its baseline correlation.

Our imputation yielded systematic improvement in concordance for both prediction directions, reflected by a widespread shift of proteins above the identity line relative to baseline (Figure 2A & Figure 2B). While our models impute the entire proteome, comparison between post-imputation and baseline correlation was restricted to the overlapping proteins. We define post-imputation correlation as Pearson correlation between machine-learning imputed platform measurement and their measured counterparts (e.g., between Olink-imputed SomaScan and measured Olink), and gains as the improvement in correlation after imputation. The mean cross-platform concordance between imputed values and their measured counterparts increased from 0.36 to 0.48 for both directions. The number of well-correlated proteins (r>0.7) doubled from ~19% to ~36–38% (N_SO_=656, N_OS_=633), and the proportion of poorly correlated proteins (r<0.4) was reduced from ~57% to 46% (N_SO_ = 801, N_OS_=790) (Figure 2A & Figure 2B).

For most proteins, imputation success (i.e., gain in correlation) relates to baseline cross-platform correlation. Proteins that were initially well-correlated between SomaScan and Olink were more amenable to accurate imputation. However, there were a number of exceptions where our models recovered signal despite poor baseline agreement. For instance, IVD (isovaleryl-CoA dehydrogenase, SO direction), which exhibited a low baseline correlation (r=0.21) between platforms, achieved a substantially higher correlation of r=0.61 after imputation, resulting in a correlation gain of 0.4. We also observed significant increases in correlation in receptor proteins in the OS direction, with several of them having moderate (r ~0.4) correlation at baseline (e.g., LEPR [leptin receptor], 0.40→0.75; TLR3 [toll-like receptor 3], 0.43→0.73).

We further evaluated model performance using the MESA held-out test set for all proteins by comparing imputed values against actual measurements (e.g., Olink-imputed SomaScan vs. actual measured SomaScan) (Figures 2C & 2D). The overall predictive performance was broadly effective for both directions (mean r=0.57), with 684 (50%) proteins exhibiting high performance, and there were 384 (22%) proteins with moderate to low performance (r<0.4, Supplementary Figure 2–3).

In addition, we examined whether protein localization influenced cross-platform concordance both at baseline and post-imputation. Proteins secreted into the blood or plasma exhibited higher baseline correlations compared to membrane-bound or intracellular proteins (Supplementary Figure 4). While all protein classes benefited from our framework, secreted and plasma proteins showed the most substantial improvements, frequently achieving moderate-to-strong imputed correlations. This suggests that the extracellular proteome may be more consistently captured across affinity-based technologies.

### Tiered Protein Reliability System with Validation and Correlation Gain

Based on the different imputation performances, we categorized the 1,737 overlapping proteins into a tiered-reliability system (Supplementary Table 2) for each training direction independently, based on conventional thresholds to interpret correlation coefficients (e.g., >0.7 is strong, <0.1 is weak or negligible)^22^. We define tier 1 as the most ‘robust’ group, with both baseline correlation and imputed concordance being r≥0.7. This high-trust subset should exhibit exceptional cross-platform portability and measurement quality, allowing researchers to replicate primary protein-outcome findings in independent cohorts using proteomics measured by either platform reliably, regardless of the use of our predictive models. In contrast, tier 4 is the ‘irreconcilable’ group, defined by having both baseline and imputed concordance r≤0.1. These proteins exhibit persistent discordance and cannot be recovered by our framework, which could be caused by platforms’ binding affinities (e.g., PILRA)^1,21^ or potential off-target binding. We also utilized enzyme-linked immunosorbent assay (ELISA) and Quanterix as gold standards to validate our categorization. In line with our tier definitions, 3 tier 1 proteins (CHI3L1 [chitinase-3-like protein 1], GDF15 [growth/differentiation factor 15], SELE [E-selectin]) were measured in the Nurses’ Health Study (NHS) and Health Professionals Follow-up study (HPFS) (N=54, Supplementary Figure 5)^23^: these proteins’ SomaScan measurements were well-correlated (Spearman correlation >0.8 for tier 1 proteins) with ELISA protein measurements. GFAP (glial fibrillary acidic protein) and NfL (neurofilament light chain) measured by SomaScan were validated to have low correlation with Alamar/Nulisa or Simoa/Quanterix (Ibanez et al., r~0.2, N=43^24^; and in Atherosclerosis Risk in Communities [ARIC], N=105, Supplemental Table 3, Supplemental Figure 6), while both SomaScan and Olink measures of MAPT (microtubule-associated protein tau) are not correlated with Simoa. These validated concordance and discrepancies support the quality of tier 1 proteins and also imply that epidemiological or clinical associations identified with tier 4 proteins could be spurious and warrant caution in findings based on proteins in this group.

After isolating tier 1 (robust) and tier 4 (irreconcilable) proteins, the remaining proteins were further categorized into two groups (tier 2: imputed r≥ 0.4, tier 3: imputed r<0.4). Tier 2 is the ‘model recoverable’ group, representing proteins with modest to low baseline correlation that gained and achieved acceptable (r>0.4) correlation post-imputation. This group expanded the ‘usable’ portion of the proteome, allowing its use in cross-platform validation. The protein IVD mentioned previously is a tier 2 protein. Lastly, the remaining proteins were categorized as tier 3, the ‘ambivalent’ group: while our imputed value offered a slight increase in concordance, the concordance remained relatively low. It is also advised that associations identified with tier 3 proteins be assessed carefully and skeptically, as their cross-platform concordance is insufficient for reproducibility.

In each direction, there are 19% tier 1, 35% tier 2, 25% tier 3, and 25% tier 4 proteins, respectively. Bi-directionally, all of tier 1 (334, 25%), and the vast majority (>90%) of tier 2 and tier 4 proteins are identical, reflecting the robustness of the membership of the tiers: tier 1 and 2 proteins are consistently reliable, and tier 4 proteins are consistently discordant. The most variability of protein tier memberships was among tier 3 proteins.

We observed clear increases in correlation across proteins in all tiers except for tier 4. Tier 1 proteins exhibited a ceiling effect, approaching a correlation of 0.93 for SO and 0.90 for OS. The most substantial gains were observed in tier 2 and some tier 3 proteins; tier 2 proteins, on average, gained 0.21 in Pearson correlation in SO direction and 0.19 in OS direction, while tier 3 proteins gained 0.08 in SO direction and 0.10 in OS direction (Figure 2A & 2B).

### Protein Importance

We annotated each protein with its ‘importance’ using two main criteria: 1) clinical utility as assessed using the Human Protein Atlas (HPA)^25,26^, classifying proteins as “FDA-approved” or “potential” drug targets, and 2) scientific attention as assessed by the MalaCards and DISEASE^27–29^ databases and by GeneALaCart to aggregate the total number of publications associated with each gene symbol.

**Figure 3.**
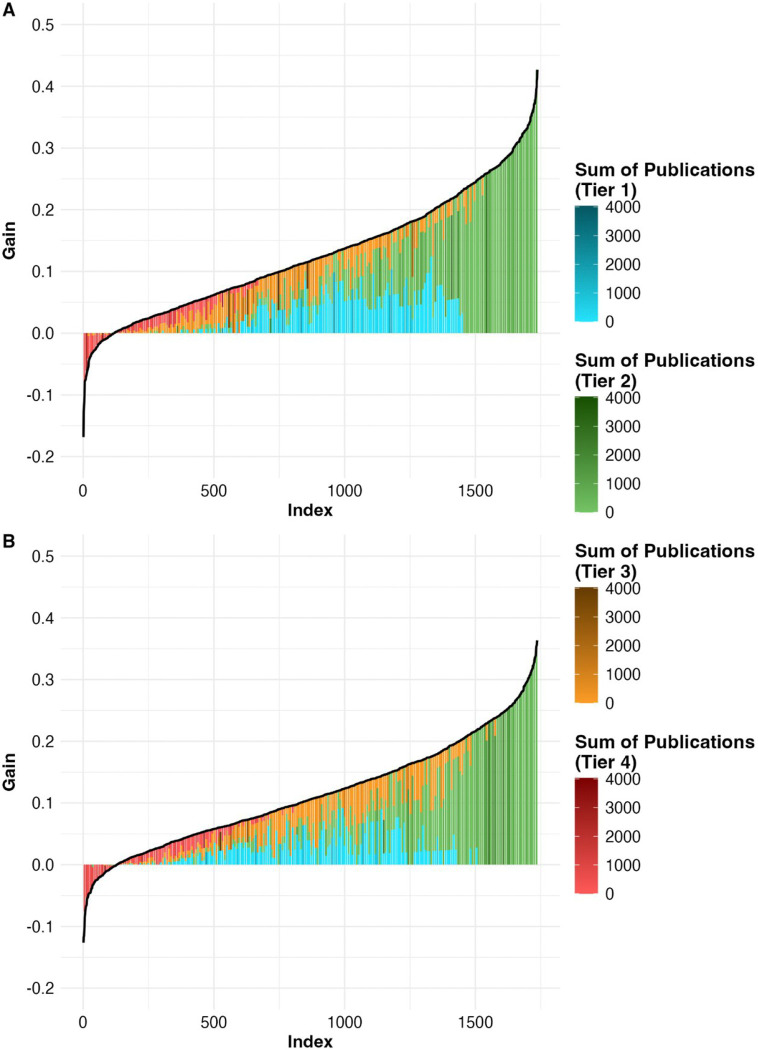
Gain in Correlation between SomaScan and Olink measurement by protein tiers. **A.** SomaScan to Olink training direction. **B.** Olink to SomaScan training direction. The Y-axis illustrates the gain in correlation (post-imputation Pearson correlation minus baseline Pearson correlation) for 1,737 overlapping proteins between the two platforms. X-axis represents the protein index, ranked in ascending order of the gain. All proteins were binned into 220 bins based on their gain for better visual demonstration of importance. Importance is defined by the sum of publications of a given protein through text mining provided by GeneCards. Within individual bins, the size of each color block (each protein tier) is proportional to the proportion of: (number of proteins in that tier / total number of proteins in the bin); the larger the proportion, the more proteins in the corresponding tier compared to other tiers present in the bin. In each bin, the aggregated number of publications of all proteins grouped by tiers is calculated: the darker the color, the more total publication proteins have and hence more important the proteins are.

**Figure 4.**
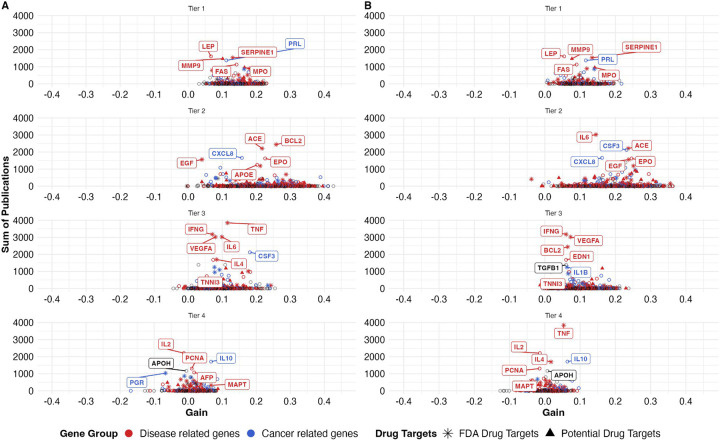
Scatter plot of the clinical relevance of 1,737 overlapping proteins. A. SomaScan to Olink direction. B. Olink to SomaScan direction. Each point represents one protein, colored by gene group (disease or cancer association), and shaped by druggability (FDA-approved or potential drug target).

Figures 3 & 4 visualized such importance of proteins stratified by our proposed tiered reliability system. Gain in correlation was not restricted to a subset of well-studied markers with a high number of associated publications visualized by darker bins (Figure 3). These ‘important’ proteins were distributed across all 4 tiers. Among the 1,737 proteins, 10% are FDA-approved drug targets, 10 % potential drug targets, 10% cancer-associated, and 34% disease associated (Figure 4). Many of the commonly discussed proteins that showed improvement in concordance, such as MPO (myeloperoxidase), were successfully identified as proteins with close overall scientific attention, having approximately or greater than 1,000 publications (Figure 4).

Some proteins that are frequently utilized in epidemiological studies were tier 4 proteins. For example, one of the proteins with the most overall scientific attention is TNF (tumor necrosis factor) in tier 4 (OS direction) or tier 3 (SO direction), and it is also an approved FDA drug target. These distributions suggested that research priority does not coincide with measurement accuracy or reliability, and some widely-used proteins (i.e., TNF, IL-6) may be measured inaccurately by one or both platforms.

### Application 1 - Tier 1 Protein for Robust and Replicable Associations

To validate our tiered reliability system, application 1 demonstrates how prioritizing tier 1 would enhance the replicability of findings. We utilized elastic net regression to model body mass index (BMI) against the 1,737 overlapping or 334 tier 1 proteins across the UKB (Olink only) and CHS (SomaScan only) cohorts, adjusting for age, sex, and race as unpenalized covariates. After removing participants with missing BMI, the final analytic dataset of UKB and CHS contained 41,185 and 3,153 participants, respectively. The mean BMI of UKB participants is 27.5 (4.8), and 26.7 (4.5) in CHS. The CHS cohort represents a geriatric US population, distinct from the middle-aged UK Biobank population (Supplemental Table 4).

**Figure 5.**
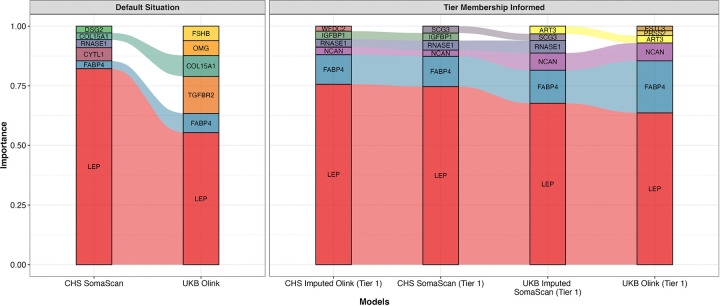
Application 1 - Feature importance of most important proteins for BMI prediction in CHS and UKB. The size of colored areas is permutation feature importance of each protein, and it is calculated using the Permutation-based approach. For each protein, we randomly permuted its values in the test data, generated new predictions using the refitted model, and calculated the resulting model predictive performance (r^2^) change as feature importance. A larger change indicates more influential proteins. The ‘default situation’ refers to situations using all 1,737 overlapping proteins, while analyses in ‘tier membership informed’ situations were restricted to using only 334 tier 1 (most robust) proteins.

A naive cross-platform analysis using the full 1,737-protein overlapping panel revealed inconsistencies in biomarker prioritization (Supplementary Table 5). Specifically, the rank-ordering of proteins with the highest predictive power for BMI showed disagreement between the UKB and CHS cohorts, with only LEP and FABP4 (fatty acid binding protein 4) exhibiting consistent importance across both platforms (Figure 5, left panel).

In contrast, prioritizing the 334 tier 1 (robust) proteins resolved the discrepancy. This subset maintained good predictive performance across both cohorts without a significant decline in overall model explanatory power (r^2^) (Supplementary Table 6). However, this tier-informed analysis selected more consistent proteins across platforms and cohorts, with or without our imputation models (Figure 5, right panel). Among the top six proteins, four proteins (LEP, FABP4, NCAN [Neurocan], and RNASE1 [ribonuclease A family member 1, pancreatic]) were successfully replicated in both cohorts for measured and machine- learning imputed platform measurements, demonstrating highly similar relative importance (Figure 5, right panel, Supplementary Table 5). These results emphasized that selecting or prioritizing tier 1 proteins significantly enhances the replicability of cross-platform epidemiological studies.

### Application 2 - Enabling Replication of Non-overlapping Proteins (Walker et al., *Nature Aging* 2021)

To evaluate the validity of imputed values, we used imputed ~5000 UKB “SomaScan” proteins based on its original Olink values to conduct epidemiological replication of previously reported protein-dementia associations by Walker et al.^30^. We selected this study because the top associated proteins were SomaScan exclusive (e.g., SVEP1 [sushi, von willebrand factor type A, epidermal growth factor, and pentraxin domain containing 1]). In brief, proteome-wide association scan was conducted using Cox proportional hazard model adjusting for covariates (Methods) identical to Walker et al.^30^. The analytical cohort for this user case was also derived from the original UKB population (Supplementary Table 1, N=41,405), with specific inclusion criteria of unrelated participants with available whole exome sequencing for apolipoprotein-E epsilon 4 (APOE-ε4) allele calling (Supplementary Table 7, N=36,854). The incidence rate of dementia per 1000 person-years in UKB was 77.3, with a median follow-up time of 13.5 years (interquartile range [IQR]: 12.8–14.3 years).

**Figure 6.**
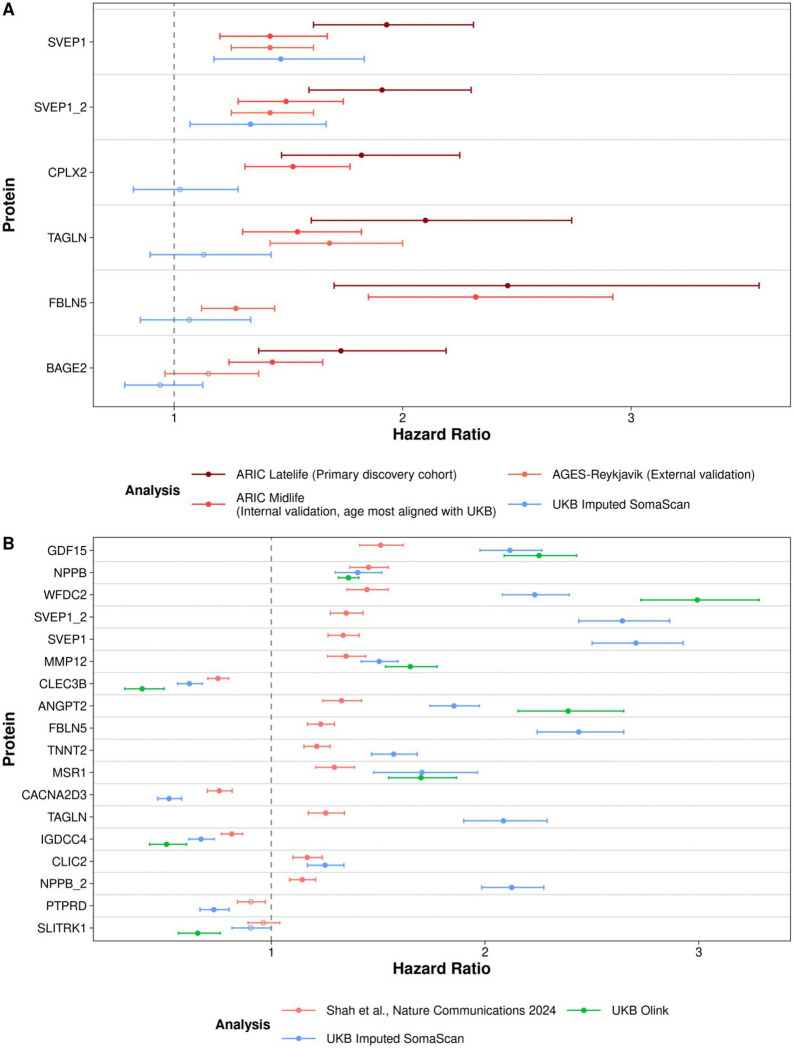
Baseline protein levels (imputed SomaScan) and the hazard of incidence dementia and heart failure in UKB (Application 2 & 3). **A. Application 2 - Association between baseline protein level and incident dementia.** Five SomaScan exclusive proteins (6 measurements) were displayed. Points are the hazard of incident dementia associated with 1-unit increase of Olink-imputed SomaScan protein level at baseline, with 95% confidence interval. Proteins are ordered according to the original study (Walker et al., Nature Aging 2021)’s study findings in A RIC latelife cohorts, with SVEP1 being most significant findings that replicated in internal validation cohort (ARIC midlife, where its mean age aligns the best with UKB’s) and in external validation cohort using AGES-Reykjavik. We successfully replicated the most important signals / findings in the original study: SVEP1. SVEP1: sushi, von Willebrand factor type A (suffix indicates values measured by different aptamers), EGF and pentraxin domain containing 1; CPLX2: complexin 2; TAGLN: transgelin; FBLN5: fibulin 5; BAGE2: B melanoma antigen 2. Each protein was associated with incident dementia in separate multivariate models adjusted for age at enrollment, sex, white British ancestry, smoking status, eGFR-creatinine, APOE-ε4, BMI, prevalent hypertension, and diabetes, identical to the original study. **B. Application 3 - Association between baseline protein level and incident heart failure.** Points are estimated hazards of incident heart failure associated with 1-imputed protein level increase with 95% confidence interval. ARIC: Atherosclerosis Risk in Communities Study; AGES- Reykjavik: The Age, Gene/Environment Susceptibility-Reykjavik; SVEP1: sushi, von Willebrand factor type A, EGF, and pentraxin domain-containing protein 1; WFDC2: WAP four-disulfide core domain protein 2; MSR1: (Macrophage Scavenger Receptor 1, or CD204; SLITRK1: SLIT and NTRK-like family, member 1; FBLN5: SLIT and NTRK-like family, member 1; TAGLN: transgelin; ANGPT2: angiopoietin-2; GFD15: growth differentiation factor 15; MMP12: matrix metallopeptidase 12; TNNT2: troponin T2; CLEC3B: c-type lectin domain family 3 member B; NPPB: natriuretic peptide B; CLIC2: chloride intracellular channel 2; CACNA2D3: calcium voltage-gated channel auxiliary subunit alpha2delta 3; PTPRD: protein tyrosine phosphatase receptor type D; IGDCC4: immunoglobulin superfamily, DCC subclass, member 4; Proteins are ordered based on most descending significance (Bonferroni adjusted p-value) in the original study (Shah et al., Nature Communications 2024).

SVEP1 is the leading discovery in the study, the only protein being highlighted in the abs tract of the paper. It was strongly associated with incident dementia in discovery using ARIC late life cohort, validated internally in ARIC midlife cohort, and externally in AGES-Reykjavik coho rt^30^. In the ARIC midlife analysis, which has the most similar mean age compared to UKB, Walker et al. demonstrated that one SD increase in the two baseline SVEP1 measurements from different aptamers on the log2 scale was associated with a 1.42-fold (95% confidence interval [CI]: 1.20–1.67) and 1.49-fold (95% CI: 1.28–1.74) increase in hazard of incident dementia. In UKB, the hazard ratio of one SD increase in baseline imputed SVEP1 value on the log2 scale and incident dementia is 1.47 (95% CI: 1.18–1.83, P=7.30 ⨉ 10^−4^) and 1.33 (95% CI: 1.07–1.66, P= 0.01), demonstrating nearly identical associations (Figure 6A, Supplementary Table 8).

### Application 3 - Improving replication of overlapping protein-outcome associations (Shah et al., *Nature Communications* 2024)

In addition to the recovery of platform-exclusive protein-phenotype associations, the third application of our imputed SomaScan values is yielding ‘more replicative and comparable’ results to SomaScan-based findings compared to using direct Olink measurements for overlapping proteins. Similar to our approach in ‘Application 2’, we adopted the same methodology as Shah et al. for our analysis (Methods)^31^. While the inclusion criteria of the UKB study population were different from ‘Application 1’ and ‘Application 2’, most of the demographic characteristics, such as mean age and BMI, remained identical (Supplementary Table 1, Supplementary Table 7). The incidence rate of heart failure per 100,000 person-years is 330.9, and the median follow-up time is 13.5 years (IQR: 12.7–14.3).

Seventeen out of 18 measurements (94%) imputed SomaScan value achieved statistical significance (P<0.05) after adjusting for multiple testing using Bonferroni. For all proteins available in both SomaScan and Olink, imputed SomaScan measurements consistently yielded estimates closer to the associations by Shah et al. than the compared to the estimates using native Olink measurements (Figure 6B, Supplemental Table 9).

### Protein Performance Interpretation with Feature Importance and Protein Structure

We further investigated the reasons behind the successes in applications and observed differences in imputed correlation. Feature importance and first K feature’s contribution to balance model performance and imputed correlation revealed that our imputation framework utilized information from protein’s biological ‘neighbors’ even when baseline correlation is high. For example, in Supplementary Table 10, FABP4 is a tier 1 protein from Application 1 and the top feature contributing to both imputed correlation and model prediction accuracy is FABP3 in SO direction and FABP4 in OS direction. While PILRA itself drove the correlation gain in magnitude in SO direction, its gains in OS direction were led by PILRB and NECTIN2, proteins that are highly correlated with PILRA^32,33^. Similarly for SERPINA5, SO imputation leveraged the strong correlated SERPINA5-F2 pair to improve performance^32,34^, a feature missed in the OS direction. When imputing SVEP1 using olink data, no protein has distinctly high feature importance (LTBP2: 0.06 [SD:0.003], CTHRC1: 0.03[SD:0.004]), but the predicitive accuracy (r^2^) of SVEP1 value reached 0.7 while LBTP2 (latent transforming growth factor beta binding protein 2) contributed 0.5 r^2^ (Supplementary Table 11)

Based on Alphafold3’s protein structure prediction, tier 1 and 2 proteins (e.g., LEP:0.11[0.01], FABP3: 0 [0]) have a lower fraction disordered compared to tier 4 proteins (PILRA: 0.60 [0.004], GFAP: 0.49 [0.03], NfL: 0.64 [0.03])

## DISCUSSION

In recent years, the exponential growth of population-scale proteomics has generated a wealth of association data. However, findings derived from one platform usually lack reproducibility when attempted on the other, one of the most frequent critiques in peer review that stifles the transition from discovery to clinical application. When non-replication occurs between characteristically different cohorts, it is often impossible to disentangle whether the failure is due to distinct environmental or biological cohort effects or simply technical platform artifacts. This uncertainty has become a rate-limiting step in the translation of epidemiological an d clinical research using proteomics, and our study is motivated by this critical bottleneck.

In this study, we leveraged a machine learning-based framework to bridge the critical gap between the two dominant high-throughput proteomic platforms, SomaScan and Olink. By leveraging paired data from MESA, we constructed bi-directional imputation models that predict full proteomic panels for both platforms. Our approach directly addresses the systemic challenge of non-replication in large-scale proteomics mentioned above, a discrepancy that largely limits the confidence of findings and thus the number of protein candidates for potential disease biomarkers and drug targets. Our framework provides a three-pronged solution: 1) a tiered protein reliability system that guides target prioritization or future assay optimization; 2) a bi-directional imputation to both recover “exclusive” proteins; and 3) the denoising and improving correlation of overlapping proteins. We demonstrated the clinical validity of this approach and its imputed values through successful replication of protein-outcome associations with BMI (application 1), incident dementia (application 2), and incident heart failure (application 3) across demographically distinct cohorts (UKB and CHS), proving that imputation can help increase replicability of cross-platform and platform-specific proteomic studies.

Previous evidence indicates that poor correlation between platforms could arise due to the epitope effect, or potential mismeasurement^1,24^. For medical and epidemiological researchers, the primary objective is to identify which reliable and robust associations are rooted either in high-fidelity assays or replicability across independent cohorts. Therefore, we chose the bi-directional approach rather than harmonizing two platforms’ measurements into one, as it may further deviate us from the underlying biology. The bi-directional imputation reconstructs the target signal by leveraging the high-dimensional proteome and learning the non-linear correlations between the target protein and its biological ‘neighbors.’ The objective of this reconstruction is also not to create ‘literal’ replications of the original measurements (e.g., tuning for high model performance/prediction accuracy), as the original measurements contain binding artifacts and noise. Our approach serves primarily as a denoising mechanism, and such imputation ensures that researchers can replicate an Olink-based discovery in a SomaScan-based cohort (or vice versa) by generating the specific counterpart values, thus validating the original signal in its native feature space. The preservation of this information would also facilitate higher replicability of epidemiological studies. One such example is SLITRK1 (tier 2, SLIT and NTRK like family member 1) from Application 3: imputed-SomaScan levels of this protein yielded a more similar point estimate than its actual Olink measures compared to the published and peer-reviewed results.

The tiered reliability system, together with the protein importance scale, offers two primary utilities for the research community. First, it enables researchers to gauge the reliability and replicability of specific findings and would potentially reduce downstream analysis costs by avoiding pursuing false positive signals. A pertinent example is the classification of GFAP (glial fibrillary acidic protein) as a tier 4 protein. This biomarker was repetitively shown to have significant discordance between SomaScan measurement and clinical gold standard measurements in multiple independent cohorts, suggesting potential binding artifact or off-target binding. Therefore, rather than immediately pursuing such signals as valid disease markers, researchers should approach findings involving tier 4 proteins with skepticism, acknowledging a higher potential for false positives. In contrast, researchers can prioritize or pursue correlations found with tier1 proteins as they exhibit strong cross-platform correlation independent of our imputation framework, and this was empirically validated by Application 1. While tier 1 and tier 4 have more defined measurement quality or model recoverability, tier 2 and 3 offered more insights into the moderate part and expanded the usable proteomes, as demonstrated in Application 3. Second, it offers different potential hypothesis testing opportunities for researchers with different research focuses, together with our quantitative protein importance scale. While downstream analyses like protein quantitative trait loci (pQTL) could prioritize tier 1 and 2 proteins, it is also worth investigating potential contributors to the persistent discordance between platforms or between platforms and clinical gold standards for tier 3 and tier 4 proteins, especially for proteins with high scientific interest. For example, unstable structures for tier 4 proteins induced by protein-altering variants predicted by Alphafold 3 could affect one or both platforms (Results).

Our machine-learning-driven model was able to identify biological signals and utilize proteins’ biological neighbors even when raw measurements appeared contradictory across platforms. For instance, PILRA is known to have SomaScan measurement bias due to binding affinity (rs1859788-A, p.G78R epitope effect), with similar suspicions regarding its Olink measurement^1,35^. While the PILRA exhibited negative baseline correlations (r=−0.4), our model achieved an increase in the magnitude of negative correlation (r=−0.50) for both SO and OS directions. However, the underlying contributing features were not the same across both directions. This highlighted that while genetic variants may modify the ‘stickiness’ of probes used by one or both platforms, some biological modification was preserved and computationally retrievable. Unlike PILRA, the different gain in magnitude of negative association (|gain_SO_|=0.17, |gain_OS_|=0.05) and different feature importance indicate a different kind of modifying effect for SERPINA5.

Not limited to the overlapping targets, our framework also enables the use of non-overlapping proteins, allowing cohorts restricted to one platform to investigate targets that were inaccessible. Application 2 suggested that imputed values can act as valid proxies for absent proteomic data, enabling accessible validation in an independent and different cohort using another platform for proteomics measurement.

Our study has limitations. First, imputed values for non-overlapping proteins remain probabilistic imputations. While our user cases demonstrate utility, these predictions are subject to error and should be interpreted with appropriate caution, particularly for proteins lacking orthogonal validation. Second, our use of histogram-based gradient boosting still minimizes for t he loss function rather than the biological mechanism. While it captures non-linear relationships, it does not physically adjust for binding artifacts, and other methods could also be used to explore this topic. To mitigate these, we validated our findings through extensive replication of peer-reviewed phenotypes in independent cohorts.

In summary, our study successfully bridges the two main proteomic measuring platforms, SomaScan and Olink, significantly enhancing the replicability of cross-platform proteomic data. By providing a public reference for protein reliability and a method to impute datasets computationally, we contribute to dismantling the barriers imposed by platform differences and potentially accelerating the translation of proteomic discoveries.

## METHODS

### Study Cohorts

#### Multi-Ethnic Study of Atherosclerosis

The Multi-Ethnic Study of Atherosclerosis (MESA) is a prospective longitudinal cohort study that recruited 6,814 men and women aged 45 to 84 years from six communities in the United States during 2000–2002^13^. The participants were free of clinically apparent cardiovascular disease at baseline and included individuals of White, Black, Hispanic, and Chinese descent. For the current analysis, plasma protein levels were measured from samples collected at Exam 1 of 5,325 participants. Proteomic profiling was performed using both the SomaScan v4.1 Panel and the Olink Explore 3072 platform.

#### Cardiovascular Health Research

The Cardiovascular Health Study (CHS) is a population-based prospective cohort study initiated to identify risk factors for the development and progression of coronary heart disease and stroke in older adults^16,17^. The study originally recruited 5,201 men and women aged 65 years and older from four U.S. communities between 1989 and 1990, with an additional cohort of 687 African American participants recruited in 1992–1993. Circulating plasma protein concentrations were assessed using the SomaScan v4.0 platform. Measurements were performed on EDTA plasma samples collected from 3,173 participants at the 1992–1993 visit, which is the baseline for the African American cohort and year 3 for the original cohort.

#### UK Biobank

The UK Biobank (UKB) is a prospective cohort study that recruited approximately 500,000 participants from the UK during 2006–2010^14,15^. This study restricted the study population to the subset of 53,018 participants with available baseline (instance 0) plasma protein measurements. The circulating plasma protein concentration was measured using Olink Explore 3072, covering up to 2923 unique protein assays per sample. Details on UKB Olink data processing have been described elsewhere^3^.

### Preparation of Protein Data

#### Data Processing of SomaScan

SomaScan utilizes SOMAmer (aptamers) reagents that bind to the target protein, and after stringent washing that removes non-specific bindings, the SOMAmer that is specifically bound to the protein is released and quantified on a standard microarray chip via fluorescent signal and returned as the value in relative fluorescent units (RFU)^5^. SomaScan data showed no missing values in either cohort. Different sets of SOMAmers targeting the same protein were kept as distinct measurements and denoted with suffixes (e.g., protein_2/3) to preserve reagent-specific reads.

Different versions of SomaScan utilize different technical processing protocols, such as reagents, well volumes, etc. As CHS utilized the SomaLogic Panel v4.0, which includes around 5,000 proteins, while MESA used the SomaScan v4.1 panel, which includes approximately 7,000 proteins, MESA data were lifted from the v4.1 panel to the v4.0 panel in this study using the SomaDataIO R package^18^. SomaDataIO is an official and open-source R package provided by SomaLogic / BioTools, Inc. to allow users to load, manipulate, and analyze data generated by SomaScan proteomic measurements. The Lifting process is a linear transformation of the raw RFU data columns between panel versions for each protein assay. The details of the lifting algorithm are well documented in the SomaDataIO R package documentation and GitHub website^18^. After lifting, the RFU values were log2 transformed, then further standardized to approximate the standard distribution of Olink NPX values.

#### Data Processing of Olink

Olink raw values were measured using PEA, and further normalized into Normalized Protein eXpression (NPX) that is proportional to the initial concentration of the plasma protein^6^. For proteins with a very low abundance or concentration in plasma, too few successful binding events would not be able to generate signals higher than the platform’s established limits of detection (LOD) threshold, and hence were marked with missing values. Proteins (columns) and participants (rows) having more than 10% missing values were excluded per quality control, and we leveraged K-nearest neighbor (KNN, R package ‘impute’ v1.62.0)^36^ imputation with 10 nearest neighbor (K=10) to impute the remaining missingness in the dataset.

Six proteins were repeatedly measured across different Olink panels, and the final protein value was chosen based on the highest target protein detectability and the largest number of NPX data points exceeding the LOD per UKB Olink processing documentation (UKB Category 1839, Resource 4654)^3^.

### Imputation of SomaScan and Olink

#### Histogram-based Gradient Boosting Regressor Algorithm

We employed a histogram-based gradient boosting regressor from the Scikit-Learn package to construct bi-direction predictive model trained on the SomaScan and Olink measurements from MESA, aiming to predict SomaScan measurements from existing Olink measurements (Olink → SomaScan (OS)) and predict Olink measurements using existing SomaScan measurements (SomaScan → Olink (SO)) for cohorts (CHS, UKB) with single-platform proteomics data.

Gradient boosting (GB) is a machine learning ensemble method that builds a predictive model. It trains a series of decision trees where each tree is explicitly designed to correct the residual errors of the previous tree^37^. This additive process minimizes a loss function, increasing the predictive ability of the model. Histogram-based gradient boosting (HGB) is a variation of this approach, optimizing computation efficiency to process large numbers of samples and features^38,39^. Instead of evaluating split points for each feature, HGB bins similar continuous input features and reduces computation complexity. It is selected for imputation as the discordance between these platforms is often non-linear and driven by different binding kinetics of the platforms. Not only can GB model non-linear relationships between proteins, but it is also robust to outliers and noise, which are common in high-throughput proteomic data due to batch effects or technical noise during and post normalization. By using these decision trees, the model can capture local variation between proteins that the global scaling methods like normalization would miss. This approach is designed to overcome issues of non-replication, therefore allowing accessible and cost-effective hypothesis testing across diverse cohorts and technologies.

For each protein measurement in the destination platform, the entire set of protein measurements from the base platform was used as input features for the histogram-based gradient boosting regressor. For SomaScan to Olink, there are 4,973 features (SomaScan proteins) and a total of 2,871 models (Olink proteins), and for Olink to SomaScan, there are 4,973 models (SomaScan proteins) with each of them utilizing 2,871 features (Olink proteins).

#### Training, Internal & External Validation

The MESA cohorts were separated into 75% training (N = 3,993) and 25% hold-out internal validation (N=1,332) sets. We calculated the baseline correlation of the overlapping proteins (N = 1,737) between SomaScan measurement and Olink measurements in the training dataset and post-imputation correlation in the hold-out internal validation set with the Pearson correlation value. In the internal validation set, the correlation between imputed protein levels and actual protein levels is also calculated using Pearson correlation coefficient. For non-overlapping proteins of each platform, only the model performance criteria (Pearson correlation) is available.

Following model training on the MESA cohort, the resulting predictive models were applied to two independent external validation cohorts (CHS and UKB). The primary objective was to assess the cross-platform performance of the imputation models for cohorts with single-platform measurement in the setting of replicating previous proteomic studies.

### Correlation Gain

We define baseline correlation as the Pearson r between log-2 transformed, scaled SomaScan RFU and Olink NPX values within the MESA training subset. Post-imputation correlation was similarly calculated in the MESA test subset, comparing machine learning-imputed values against their actually measured counterparts (e.g., imputed SomaScan versus measured Olink). The resulting correlation gain represents the difference between these post-imputation and baseline Pearson r values.

### Tiered Protein Reliability System

Protein tiers were defined based on the baseline and post-imputation Pearson correlation coefficients (r): 1) tier 1 proteins were categorized if they had both baseline and post-imputation r ≥0.7; 2) tier 4: both baseline and post-imputation r<0.1.

After defining tier 1 and 4 proteins, the remaining proteins were further categorized based on their post-imputation correlation. If the post-imputation correlation ≥0.4, it will be in tier 2; otherwise (r <0.4), it will be in tier 3 (Supplementary Table 2).

### Protein Importance

The importance of a protein is evaluated through 2 main criteria, clinical relevance and overall scientific attention, across two sources: the Human Atlas of Proteins (HPA)^25,26^ and MalaCards, the human disease database from the GeneCards Suite^27^. HPA highlights a protein’s established drugability and clinical relevance by indicating whether this protein is an “FDA approved drug target” or a “potential drug target” in its “Protein Class” section. On the other hand, we quantify the overall scientific attention of a protein by the sum of relevant publications of that gene symbol or protein name in the MalaCards database. A previous publication had described the data mining and database-building process of MalaCards in detail^27^. In Brief, MalaCards searched the protein and/or gene name in databases like DISEASE^28,29^, Novoseek, or PubMed to establish the association between a protein and/or gene with a specific publication. We curated the information from MalaCards using GeneALaCart and calculated the aggregated sum of publications associated with a protein or gene symbol.

We interpret the protein importance as the following: 1) FDA-approved drug targets or potential drug targets have more clinical relevance than proteins that have no available information in this criterion, and 2) the higher the total number of publications, the more overall scientific attention it receives and hence the more important to the research community.

### Application of Imputed Protein Measurements

#### Application 1 – Tier 1 Protein for Consistent Associations (BMI as example)

We associate all the proteins with Body Mass Index (BMI), a common continuous phenotype in both CHS and UKB, using all proteins from their actual protein measurements (CHS: SomaScan, UKB: Olink) and tier 1 proteins of the actual measurements and imputed measurements, controlling for age, sex, and race. For CHS and UKB, the predictive modeling utilized elastic net regression using the “glmnet” R package to assess the association between the protein features and BMI individually. To ensure optimal performance of feature selection and elastic net, the mixing parameter ⍺ and regularization parameter ƛ were tuned on the full cohort using 10-fold cross-validation. Unpenalized covariates (age, sex, and race) were included and assigned a penalty factor of 0. The final model (UKB, CHS separately) was trained on the 80% split of the training data using the optimized tuning parameter and evaluated on the remaining 20% hold-out test set, and the model performance is measured by the square of Pearson r between predicted BMI using the model and actual measurement of BMI (r^2^). The feature importance of each protein was then quantified using the permutation feature importance (PFI) on the test set, where the importance of a protein to the model prediction was measured by the drop in r^2^ after randomly shuffling that protein’s values.

#### Application 2 – Enabling Replication of Non-overlapping Proteins (Walker et al., Nature Aging 2021)

We also conducted a replication proteomic study of the findings reported by Walker et. al., which utilized the SomaScan v4.0 platform^30^. Separate from the tier membership informed analysis, the primary goal of this replication is to demonstrate the performance of non-overlapping proteins between SomaScan and Olink, a common obstacle faced when replicating study findings across cohorts. Briefly, Walker et al. leverage Atherosclerosis Risk in Communities (ARIC) cohort’s data from visit 5 (late-life) as discovery, and validated their findings internally in ARIC midlife (visit 3) and externally in the AGES-Reykjavik study. They utilized Cox proportional hazard regression model to examine the association between the standardized level of proteins (SomaScan) and the incidence of dementia, controlling for age, sex, race-center, education (Supplemental Table 6), APOE-ε4, eGFR-creatinine^40^, BMI, prevalent cases of diabetes, hypertension, and smoking status, as their primary analysis methods. To ensure methodological consistency, we harmonized definitions of covariates, exposures, and outcomes definition with the original analysis. Due to the absence of protocol-based dementia and cognitive assessment in UKB, we approximated dementia phenotype with available ICD-9 and ICD-10 codes from self-report and HESIN records.

UKB is more phenotypically similar to ARIC midlife in terms of mean age. Therefore, the goal of replication is to replicate the found associations in ARIC midlife replication in UKB using imputed SomaScan measures. Prior to the association analysis using Cox proportional hazard model, KNN imputation (k=10) was used to impute missing covariates.

#### Application 3 - Improving Cross-platform Protein Association Replication. (Shah et. al., Nature Communication 2024)

Additionally, we performed another replication study of Shah et al.’s 2024 study on proteomic association with incidence of heart failure^31^. In summary, their study cohort also utilized ARIC midlife and late-life visits, coupled with an external validation cohort of the Trøndelag Health (HUNT) Study. Shah et. al. also leveraged Cox proportional hazard model to assess the association between each individual protein and incidence of Heart Failure, adjusting for age, sex, BMI, eGFR based on creatinine (CKD-EPI), race, sex, smoking status, and prevalent cases of coronary artery disease, diabetes, atrial fibrillation, and hypertension. Likewise, we performed the replication analyses with the same set of covariates, exposures, and phenotypes (ICD codes).

##### Feature Importance

For feature mentioned in Application 2 and 3, feature importance is calculate using permuted importance in sklearn with five repeats. The permuted feature importance calculated the differences in R2 after randomly shuffling a feature’s values, and the mean and standard deviation of the feature importances across the 5 repeats were calculated for each feature. Then, features are arranged according to descending order of the mean importance, and the first K features (K=1,2,3,4,5,50,100,…,300) are used to retrain the model to determine which feature contribute to the model performance (imputed and actual value) and imputed correlation (e.g. SomaScan and SomaScan imputed Olink in overlapping proteins).

#### Alphafold 3

We used AlphaFold 3 to predict protein structures as further validations. The canonical FASTA sequences was retrieved from UniProt and then submitted to the Alphafold3 server, performing multiple runs for each protein. For each outputted JSON files, features like ranking score, pTM, chain pTM mean, chain pair iPTM mean, chain paired PAE min mean, and fraction disordered are outputted. Methods in calculating these features have been described in the Alphafold3 liteature^41^. For each protein, we averaged the metrics and analyzed them to identify structural factors linked to tier stratification.

## Supplementary Material

Supplement 1

## Data Availability

Downloadable protein information data can be found on the Human Protein Atlas website (https://www.proteinatlas.org/about/download). MalaCards for genes and proteins for academic use can be used with the GeneALaCart tool within the GeneCards Suite at https://genealacart.genecards.org/. Individual-level data from the Trans-Omics for Precision Medicine (TOPMed) program are available upon submission of a research proposal to the Data Access Committee through the Database of Genotypes and Phenotypes (dbGaP) at https://dbgap.ncbi.nlm.nih.gov/. Individual-level data from the UK Biobank are available to registered researchers via the UK Biobank Access Management System (https://www.ukbiobank.ac.uk) with an approved application; this study was conducted under application number 7089.

## Code Availability

https://github.com/zhiyulab/Cross-platform-proteomic-imputation
