# Supplementary material for: Machine learning cross-platform proteomic imputation enables protein quality scoring and replication of epidemiological associations
Source: bioRxiv. 2026 May 9:2026.05.05.723059. Preprint. [Version 1] doi: 10.64898/2026.05.05.723059 (PMC13174605; doi:10.64898/2026.05.05.723059)
Supplement: Supplement 1 [file media-1.pdf]

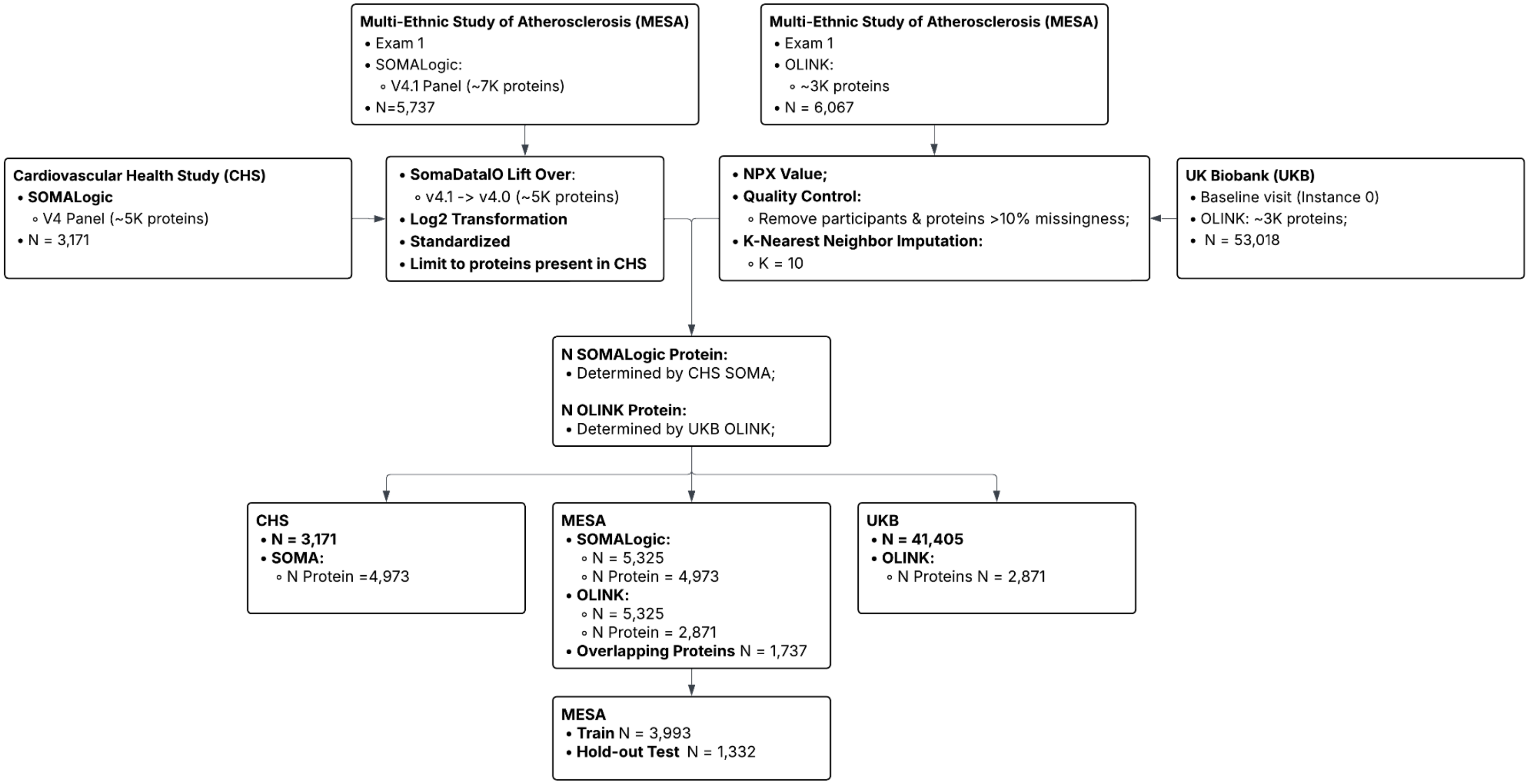

Supplementary Figure 1. Consort diagram of sample and protein quality control in three cohorts: MESA, UKB, and CHS. MESA: Multi-Ethnic Study of Atherosclerosis; UKB: UK Biobank; CHS: Cardiovascular Health Study.

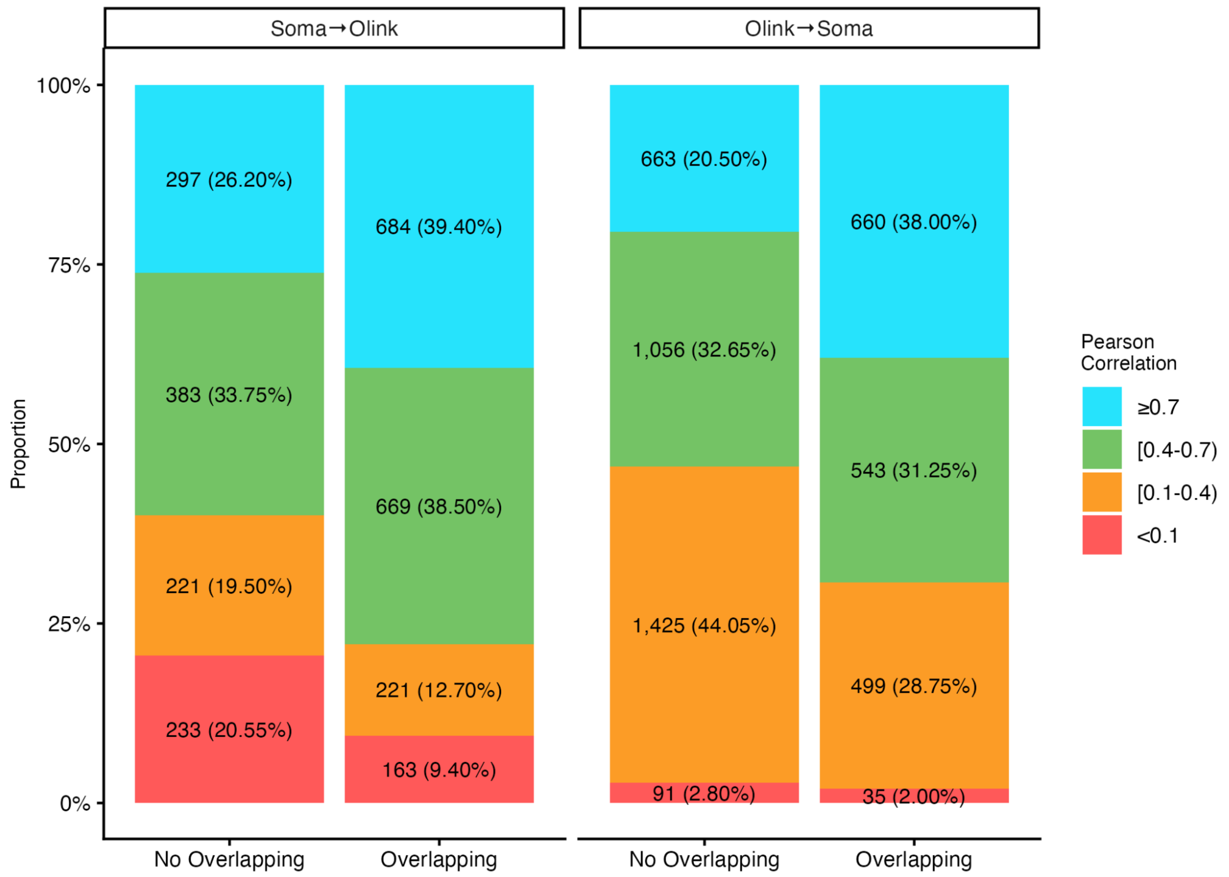

Supplementary Figure 2. Model predictive performance (Pearson correlation) in the MESA held-out test set. Model performance is calculated between the model-imputed value and the actual measured value. The size of the color block is proportional to the percentage of each Pearson correlation category. MESA: Multi-Ethnic Study of Atherosclerosis.

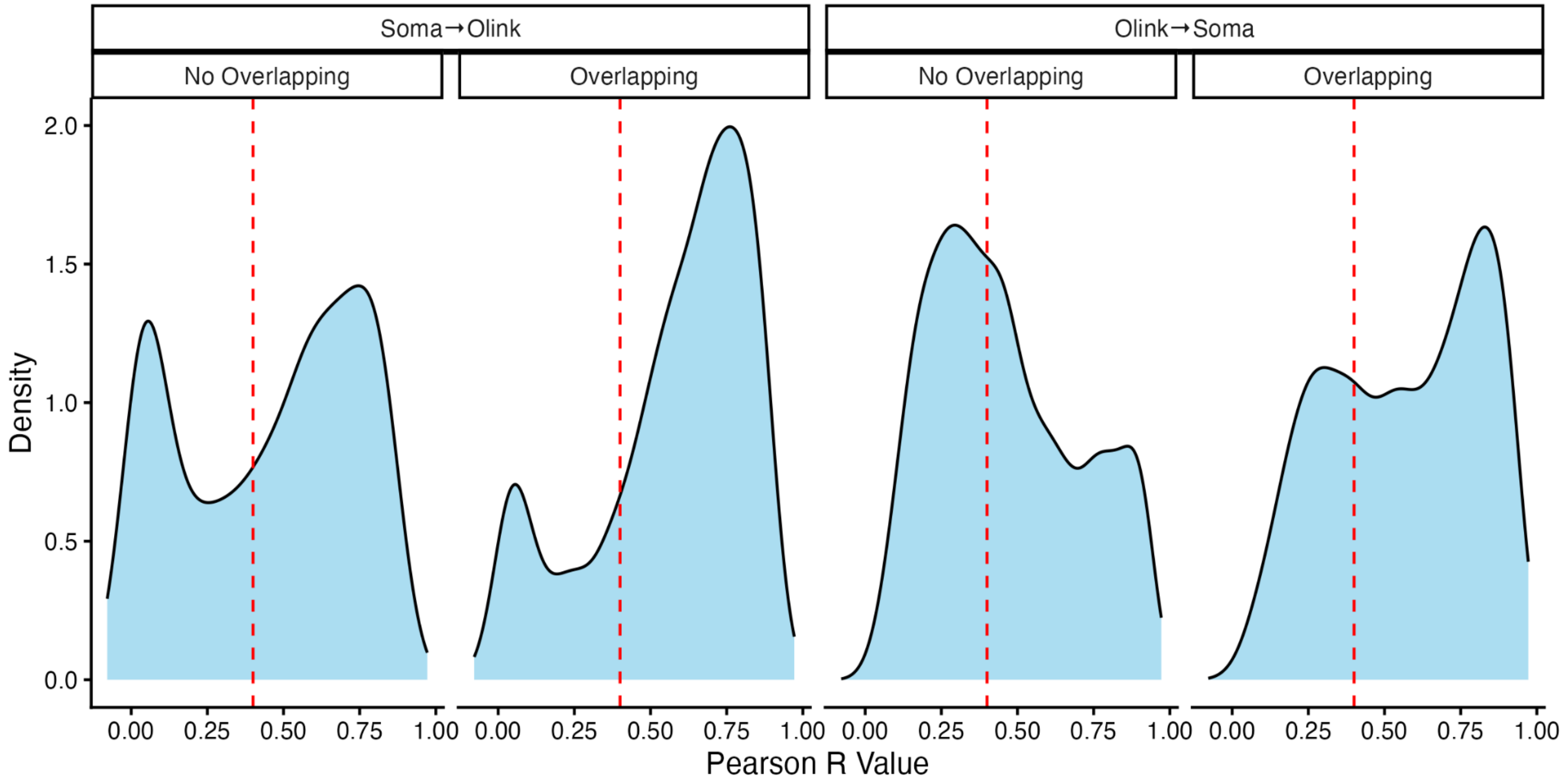

Supplementary Figure 3. Distribution of model performance (Pearson correlation [r]) in the MESA hold-out test set. The red dotted line represents r=0.4, and the black solid lines represent the density of a given model performance (r). Model performance is calculated between the model-imputed value and the actual measured value. MESA: Multi-Ethnic Study of Atherosclerosis.

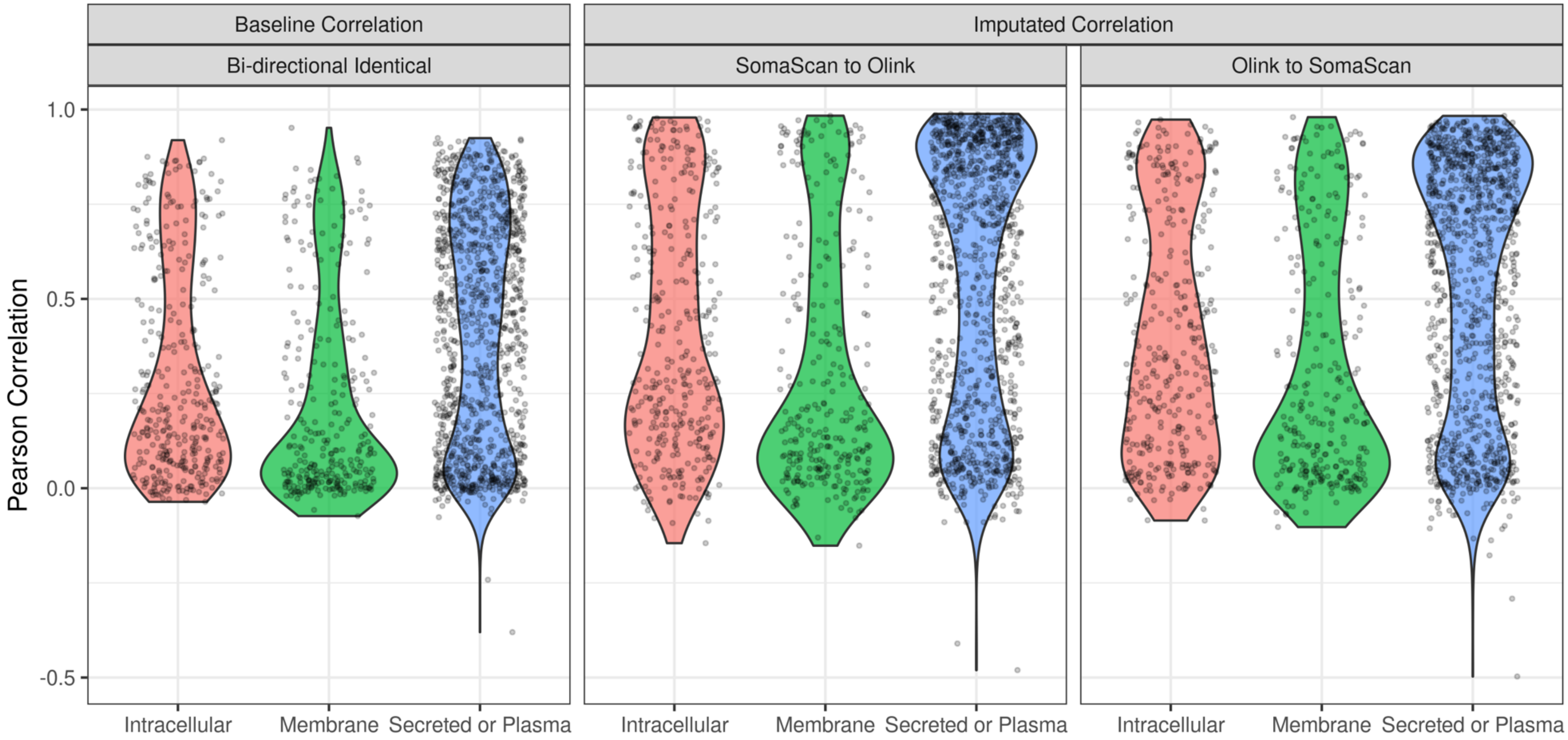

Supplementary Figure 4: Correlation between two platforms by protein localization. Proteins are classified as intracellular, membrane, secreted or plasma, as this study utilized plasma proteomic datasets based on Human Protein Atlas's protein class information. Violin plots display the distribution of correlations between the two platforms. Baseline correlation is identical across both directions. Imputed correlation was calculated using SomaScan and SomaScan-imputed Olink, or Olink and Olink-imputed SomaScan. Plasma or proteins secreted into the plasma benefited the most from imputation as the model reconstructed their measurement value with information from their biological 'neighbors'.

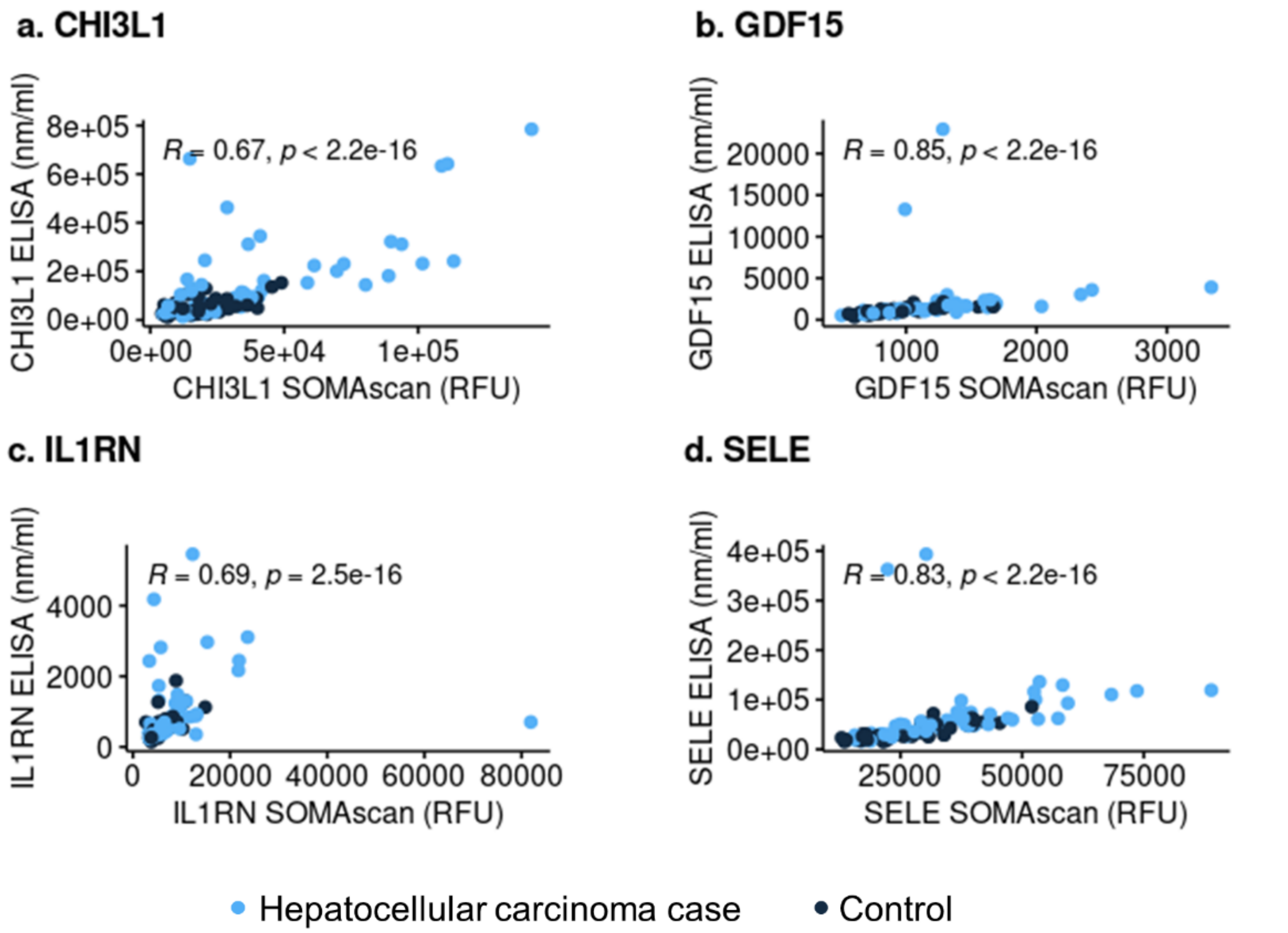

Supplementary Figure 5. Spearman correlations between the SomaScan and ELISA protein levels in the Nurses' Health Study and Health Professionals Follow-up Study (N=54).

Re-use with permission from lead author Xinyuan Zhang. Figure also published as Supplemental Figure 2 in Zhang et al., Journal of the National Cancer Institute 2024, doi: 10.1093/jnci/djae079.

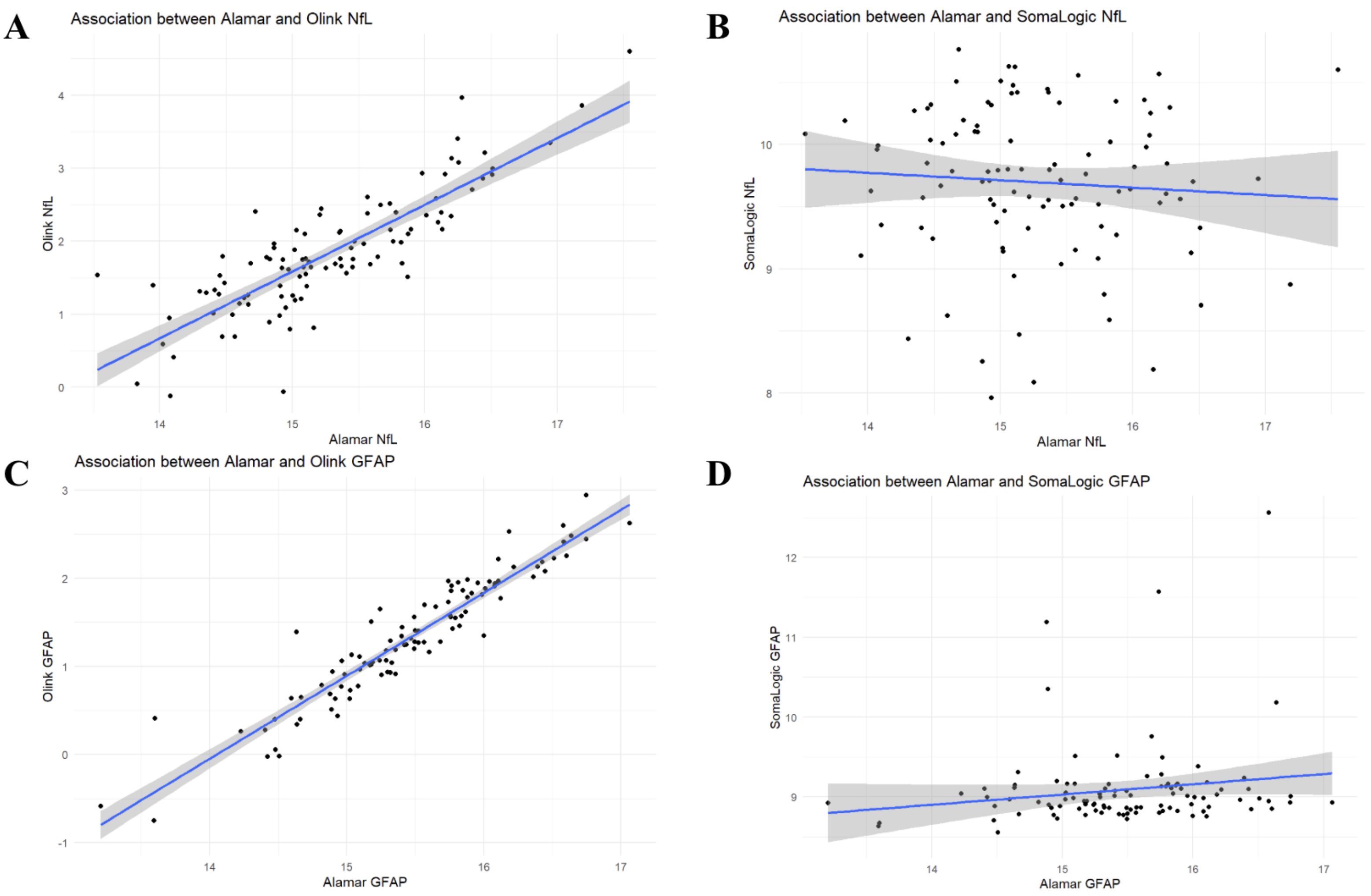

Supplementary Figure 6: Spearman Correlation between Alamar, SomaScan and Olink measurements in Atherosclerosis Risk in Communities (N=101)

Correlation calculated between Alamar and SomaScan and Olink (N~101). The Simoa (ELISA based, gold standard) had a high correlation with Alamar assays (>0.9 after excluding 1 outlier)

NfL: neurofilament light polypeptide; GFAP: glial fibrillary acidic protein;

Supplementary Table 1. Demographics of the 3 study cohorts (MESA, UKB, CHS)[1]

Metrics[2]	MESA (N=5,325)	UKB (N=41,405)	CHS (N=3,171)
**Age (years)**	62.2 (10.3)	57.3 (8.19)	74.4 (4.91)
**Sex**			
Female	2,763 (51.9%)	22,389 (54.1%)	1,927 (60.8%)
Male	2,562 (48.1%)	19,016 (45.9%)	1,244 (39.2%)
**Race**			
White	2,111 (39.6%)	38,539 (93.1%)	2,649 (83.5%)
Black	1,349 (25.3%)	1,009 (2.4%)	505 (15.9%)
Hispanic/Latino	1,223 (23.0%)	-	-
Chinese	642 (12.1%)	123 (0.3%)	-
South Asian	-	772 (1.9%)	-
American Indian /Alaskan Native	-	-	4 (0.1%)
Asian / Pacific Islander	-	-	3 (0.1%)
Other	-	962 (2.3%)	10 (0.3%)

[1]MESA: Multi-Ethnic Study of Atherosclerosis; UKB: UK Biobank; CHS: Cardiovascular Health Study.

[2]Metrics are presented as mean (standard deviation) for continuous variables, and n (%) for categorical variables.

Supplementary Table 2. Protein Tier membership thresholds

Tiers	Baseline correlation (r)[1]	Imputed correlation (r)
Tier 1, 'most robust'	≥0.7	≥0.7
Tier 2, 'model recoverable'		≥0.4
Tier 3, 'ambivalent'		<0.4
Tier 4, 'irreconcilable'	<0.1	<0.1

[1]Correlation defined as Pearson correlation. Baseline correlation is defined as r between the measured SomaScan and Olink value. Imputed correlation is defined as the Pearson correlation between SomaScan and SomaScan-imputed Olink (SomaScan to Olink direction), or between Olink and Olink-imputed SomaScan (Olink to SomaScan direction).

Supplementary Table 3. Alamar validation MAPT, GFAP, and NfL in the Atherosclerosis Risk in Communities Study.

	Alamar - Simoa Correlation[1]	Alamar Correlation[1]			Simoa / Quanterix Correlation[1]		
**Protein[2]**	(N=14)	**N**	**with SomaScan**	**with Olink**	**N**	**with SomaScan**	**with Olink**
**NfL**	0.86	101	-0.11	0.81	14	-0.14	0.76
**GFAP**	0.67	101	0.14	0.94	14	0.19	0.54
**MAPT / pTau 181**	0.87	101	-0.01	0.07	14	0.2	-0.11

[1]Correlation: Spearman correlation; correlation calculated between Alamar and SomaScan and Olink (N=101).

[2]NfL: neurofilament light polypeptide; GFAP: glial fibrillary acidic protein; MAPT: microtubule-associated protein tau;

Supplementary Table 4. Demographic characteristics of BMI analytical cohorts

Metrics[1]	UKB[2] (N=41,185)	CHS[2] (N=3,153)
**BMI**	27.4 (4.80)	26.7 (4.54)
**Age (years)**	57.3 (8.19)	74.4 (4.90)
**Sex**		
Female	22,294 (54.1%)	1,914 (60.7%)
Male	18,891 (45.9%)	1,239 (39.3%)
**Race**		
White	38,370 (93.2%)	2,633 (83.5%)
Black	994 (2.4%)	503 (16.0%)
Hispanic/Latino	-	-
Chinese	123 (0.3%)	-
South Asian	756 (1.8%)	-
American Indian /Alaskan Native	-	4 (0.1%)
Asian / Pacific Islander	-	3 (0.1%)
Other	942 (2.3%)	10 (0.3%)

[1]Metrics are presented as mean (standard deviation) for continuous variables, and n (%) for categorical variables. BMI: body mass index (Kg/m^2), calculated using weight (Kg) / $height^2$ (m).
[2]UKB: UK Biobank; CHS: Cardiovascular Health Study.

Supplementary Table 5. Application 1 - feature importance of proteins in BMI prediction

CHS SomaScan	UKB Olink	CHS Imputed Olink (Tier 1)	CHS SomaScan (Tier 1)	UKB Imputed SomaScan (Tier 1)	UKB Olink (Tier 1)
LEP (0.335)	LEP (0.078)	LEP (0.368)	LEP (0.422)	LEP (0.270)	LEP (0.270)
CYTL1 (0.022)	TGFBR2 (0.022)	FABP4 (0.06)	FABP4 (0.072)	FABP4 (0.056)	FABP4 (0.093)
RNASE1 (0.014)	COL15A1 (0.012)	IGFBP1 (0.016)	RNASE1 (0.023)	NCAN (0.029)	NCAN (0.032)
FABP4 (0.014)	FABP4 (0.011)	RNASE1 (0.016)	IGFBP1 (0.018)	RNASE1 (0.021)	ART3 (0.013)
COL15A1 (0.012)	OMG (0.009)	NCAN (0.016)	SCG3 (0.016)	ART3 (0.013)	PRSS2 (0.009)
DSG2 (0.012)	FSHB (0.009)	WFDC2 (0.01)	NCAN (0.015)	SCG3 (0.011)	FSTL3 (0.008)

[1]Feature importance calculated with permutation feature importance, where the importance of a protein to the model prediction was measured by the drop in r^2 after randomly shuffling that protein's values.

Supplementary Table 6. R-square of BMI-related Analyses[1]

	**Olink**	**Tier1 Olink**	**Tier1 SomaScan**
**UKB**	0.803	0.729	0.727
	**SomaScan**	**Tier1 SomaScan**	**Tier1 Olink**
**CHS**	0.717	0.680	0.667

[1]R-square calculated by squaring the Pearson correlation between predicted and actual BMI.

Supplementary Table 7. Characteristics of the study population in the UK Biobank in replication studies

**Application 2 - Walker et al., Nature Aging 2021**		**Application 3 - Shah et al., Nature Communications 2024**	
**Metrics(Dementia)²**	**Dementia (N=36,854)**	**Metrics (Heart Failure)**	**Heart Failure (N=41,071)**
**Age (years)**	57.3 (8.16)	**Age (years)**	57.4 (8.18)
**Sex**		**Sex**	
Female	19,020 (53.9%)	*Female*	21,229 (53.9%)
Male	16,283 (46.1%)	*Male*	18,131 (46.1%)
**White British**	29,308 (83.0%)	**White British**	32,576 (82.8%)
**BMI**	27.4 (4.72)	**BMI**	27.4 (4.73)
**Ever Smoker**	16,158 (45.8%)	**Ever Smoker**	17,953 (45.6%)
**eGFR-Creatinine**[2]	93.8 (14.0)	**eGFR-Creatinine**[2]	93.9 (13.6)
**Baseline Hypertension**	11,653 (33.0%)	**Baseline Hypertension**	13,061 (33.2%)
**Prevalent Diabetes**	2,283 (6.5%)	**Prevalent Diabetes**	2,571 (6.5%)
**APOE-ε4**[3]	10,289 (29.1%)	**Prevalent CAD**[5]	2,572 (6.5%)
**Education**[4]		**Prevalent AF**[5]	889 (2.3%)
≤High School	15,769 (44.7%)		
Vocational School	2,313 (6.6%)		
General Education	3,889 (11.0%)		
Graduate or Professional School	13,332 (37.8%)		

[1]Dementia: Dementia, Walker et. al., Nature Aging 2021; Heart Failure: Shah et. al., Nature Communications 2024.
[2]eGFR-Creatinine: estimated glomerular filtration rate, calculated using the 2021 CKD-EPI Creatinine equation.
[3]APOE-ε4: Apolipoprotein E ε4 status, presence of at least one ε4 allele.

[4]Education: Less than and high school: O levels/GCSEs or equivalent, CSEs or equivalent, and 'none of the above' option. Vocational School: NVQ, HND, HNC or equivalent. General Education: A levels/AS levels or equivalent. Graduate or Professional School: College or University degree, Other professional qualifications, e.g., nursing, teaching.
[5]CAD: Coronary Artery Disease; AF: Atrial fibrillation or flutter.
Metrics are presented as mean (standard deviation) for continuous variables, and n (%) for categorical variables. The study population was restricted to unrelated individuals in the UK Biobank with available whole-exome sequencing for the Dementia replication study, with unrelatedness defined as less than third-degree relatedness. No such exclusion for the HF study, as it didn't utilize genetic information. For individual analysis on dementia and HF, the study populations were further restricted to individuals free of the outcome disease at baseline.

Supplementary Table 8. Application 2 - Replication Results of Walker et al., Nature Aging 2021

	Walker et. al., Nature Aging 2021 (ARIC midlife)		UKB Predicted SomaScan		Walker et. al., Nature Aging 2021 (ARIC latelife)		Walker et. al., Nature Aging 2021 (AGES-Reykjavik)	
**Protein**	**HR (95% CI)**	**P-value**	**HR (95% CI)**	**P-value**	**HR (95% CI)**	**P-value**	**HR (95% CI)**	**P-value**
SVEP1	1.42 (1.20-1.67)	3.05E-05	1.47 (1.17-1.83)	7.40E-04	1.93 (1.61-2.31)	7.03E-13	1.42 (1.25-1.61)	8.17E-08
SVEP1_2	1.49 (1.28-1.74)	3.43E-07	1.33 (1.07-1.66)	1.07E-02	1.93 (1.59-2.30)	3.94E-12	1.42 (1.25-1.61)	2.82E-08
CPLX2	1.52 (1.31-1.77)	3.03E-08	1.03 (0.82-1.28)	8.24E-01	1.82 (1.47-2.25)	4.08E-08	(not measured)	
TAGLN	1.54 (1.30-1.82)	7.36E-07	1.13 (0.90-1.42)	3.06E-01	2.10 (1.60-2.74)	5.84E-08	1.68 (1.42-2.00)	3.85E-09
FBLN5	2.32 (1.85-2.92)	4.24E-13	1.07 (0.85-1.34)	5.74E-01	2.46 (1.70-3.56)	1.84E-06	1.27 (1.12-1.44)	2.00E-04
BAGE2	1.43 (1.24-1.65)	1.11E-06	0.94 (0.78-1.13)	4.97E-01	1.73 (1.37-2.19)	5.32E-06	1.15 (0.96-1.37)	1.40E-01

[1]HR: hazard ratio.

Proteins are ordered according to the original study (Walker et al., Nature Aging 2021)'s significance, with SVEP1 being most significant findings that replicated across cohorts in observational studies and in causal inferencing using (two-sample Mendelian randomization.

Supplementary Table 9. Replication Results of Shah et al., Nature Communications 2024

	Shah et al., Nature Communications 2024		UKB Imputed SomaScan		UKB Olink	
**Protein**	**HR (95% CI)**	**P-value**	**HR (95% CI)**	**P-value**	**HR (95% CI)**	**P-value**
GDF15	1.51 (1.41-1.62)	2.73E-33	2.12 (1.98-2.27)	6.66E-102	2.25 (2.09-2.43)	1.94E-99
NPPB	1.46 (1.37-1.55)	1.75E-32	1.40 (1.30-1.52)	9.58E-18	1.36 (1.31-1.41)	3.32E-67
WFDC2	1.45 (1.35-1.55)	1.69E-27	2.23 (2.08-2.39)	3.52E-112	2.99 (2.73-3.28)	4.18E-120
SVEP1_2	1.35 (1.28-1.43)	2.23E-25	2.64 (2.44-2.86)	3.86E-124	-	-
SVEP1	1.34 (1.27-1.41)	2.58E-25	2.71 (2.50-2.93)	1.28E-135	-	-
MMP12	1.35 (1.26-1.44)	4.45E-19	1.50 (1.42-1.59)	1.94E-44	1.65 (1.53-1.78)	3.68E-41
CLEC3B	0.75 (0.70-0.80)	7.12E-18	0.62 (0.56-0.68)	1.13E-23	0.40 (0.31-0.50)	3.60E-15
ANGPT2	1.33 (1.24-1.42)	4.08E-16	1.85 (1.74-1.97)	1.95E-84	2.39 (2.16-2.65)	1.72E-61
FBLN5	1.23 (1.17-1.30)	1.96E-15	2.44 (2.24-2.65)	2.19E-98	-	-
TNNT2	1.21 (1.15-1.28)	6.49E-14	1.57 (1.47-1.68)	2.57E-39	-	-
MSR1	1.30 (1.21-1.39)	4.81E-13	1.70 (1.48-1.96)	1.73E-13	1.70 (1.55-1.87)	6.23E-29
CACNA2D3	0.76 (0.70-0.82)	1.13E-12	0.52 (0.47-0.58)	1.23E-32	-	-
TAGLN	1.26 (1.17-1.34)	3.59E-11	2.09 (1.90-2.29)	8.30E-54	-	-
IGDCC4	0.82 (0.77-0.87)	5.09E-11	0.67 (0.61-0.73)	1.20E-18	0.51 (0.43-0.60)	7.15E-15
CLIC2	1.17 (1.10-1.24)	2.43E-07	1.25 (1.17-1.34)	9.57E-11	-	-

NPPB_2	1.15 (1.09-1.21)	5.29E-07	2.12 (1.98-2.28)	1.78E-103	-	-
SLITRK1	0.96 (0.89-1.04)	3.36E-01	0.90 (0.82-1.00)	4.96E-02	0.66 (0.57-0.76)	2.59E-08
PTPRD	0.90 (0.84-0.97)	6.35E-03	0.73 (0.67-0.80)	3.65E-11	-	-

[1]HR: hazard ratio.

Supplementary Table 10. Feature importances of 3 selected proteins (Top 10 features)

FABP4 (fatty acid-binding protein 4)					
**SomaScan to Olink**			**Olink to SomaScan**		
**Feature**	**Mean**	**SD**	**Feature**	**Mean**	**SD**
FABP3	1.5884	0.0384	FABP4	0.8697	0.0341
SIGLEC7	0.0014	0.0007	PALM	0.0041	0.0007
EEF2K	0.0006	0.0003	EGFR	0.0030	0.0009
DSC2	0.0005	0.0002	EDA2R	0.0026	0.0008
WISP2	0.0005	0.0002	ITGB2	0.0026	0.0005
SCO1	0.0005	0.0003	NAGPA	0.0025	0.0006
MMP10	0.0004	0.0001	CLMP	0.0025	0.0010
PLG	0.0003	0.0002	CALB2	0.0018	0.0004
H1FX	0.0003	0.0003	THOP1	0.0016	0.0009
TMEM132D	0.0003	0.0002	RGMA	0.0014	0.0000
**PILRA**					
**SomaScan to Olink**			**Olink to SomaScan**		
**Feature**	**Mean**	**SD**	**Feature**	**Mean**	**SD**
PILRA	0.2415	0.0147	PILRB	1.2681	0.0116
TNFRSF1B_2	0.1502	0.0066	NECTIN2	0.0299	0.0034
PILRA_2	0.1158	0.0051	TNFRSF1B	0.0201	0.0023
TNFRSF1A	0.0216	0.0054	IL18BP	0.0193	0.0024
CD300C	0.0056	0.0012	RNASET2	0.0104	0.0012
IL18BP	0.0041	0.0008	CD300C	0.0067	0.0023
CD48	0.0029	0.0012	FCAR	0.0057	0.0014

LILRA5_2	0.0024	0.0005	DSC2	0.0048	0.0014
CD5L	0.0023	0.0010	CLEC4D	0.0037	0.0004
MSR1_2	0.0022	0.0004	IL12RB1	0.0037	0.0002
**SERPINA5 (serpin family A member 5)**					
**SomaScan to Olink**			**Olink to SomaScan**		
**Feature**	**Mean**	**SD**	**Feature**	**Mean**	**SD**
CDH3	0.0708	0.0063	SPINT2	0.2289	0.0160
F2	0.0561	0.0018	TGFBR3	0.1305	0.0098
KNG1_2	0.0304	0.0034	ADAMTS15	0.0953	0.0109
IL2	0.0082	0.0012	GDF2	0.0408	0.0074
ICT1	0.0068	0.0031	WAS	0.0314	0.0045
PLXDC1	0.0034	0.0007	NPY	0.0241	0.0026
RBP4	0.0028	0.0003	RAB6A	0.0135	0.0019
CDH15	0.0025	0.0020	IGLC2	0.0078	0.0067
BCHE	0.0022	0.0003	CD4	0.0059	0.0066
SERPINA5	0.0018	0.0003	SERPINI1	0.0059	0.0006
**SVEP1(sushi, von Willebrand factor type A, EGF and pentraxin domain containing 1)[1]**					
**SomaScan to Olink**			**Olink to SomaScan**		
**Feature**	**Mean**	**SD**	**Feature**	**Mean**	**SD**
			LTBP2	0.0646	0.0033
			CTHRC1	0.0261	0.0036
			ADM	0.0191	0.0027
			MAMDC2	0.0152	0.0027

			THBS2	0.0104	0.0016
			SPON1	0.0086	0.0026
			CCDC80	0.0085	0.0040
			FSTL1	0.0075	0.0015
			TTR	0.0058	0.0012
			COL15A1	0.0055	0.0046

[1]For SVEP1, there's no SomaScan to Olink measurement as it is a SomaScan exclusive protein;
Feature importance calculated using "permutation_importance" function in sklearn, with scoring as r^2 between model predicted value and the actual value in the MESA test set. For each protein, n_resample = 5, and the mean and standard deviation of the feature importance are recorded.

Supplementary Table 11. K-feature model performance and imputed correlation

	FABP4 (fatty acid-binding protein 4)			
	SomaScan to Olink		Olink to SomaScan	
K Features	Model Performance	Imputed Correlation	Model Performance	Imputed Correlation
1	0.89	0.92	0.82	0.99
2	0.89	0.92	0.84	0.96
3	0.89	0.92	0.86	0.94
4	0.89	0.91	0.87	0.93
5	0.89	0.90	0.87	0.93
7	0.89	0.90	0.87	0.93
10	0.89	0.90	0.88	0.92
15	0.89	0.90	0.89	0.92
20	0.89	0.90	0.88	0.91
30	0.89	0.90	0.89	0.91
40	0.89	0.90	0.89	0.91
50	0.89	0.90	0.89	0.91
70	0.89	0.90	0.89	0.91
100	0.89	0.90	0.89	0.91
150	0.89	0.90	0.89	0.91
200	0.89	0.90	0.89	0.91
300	0.89	0.90	0.89	0.91
	PILRA (paired immunoglobin like type 2 receptor alpha)			
	SomaScan to Olink		Olink to SomaScan	
K Features	Model Performance	Imputed Correlation	Model Performance	Imputed Correlation

1	0.48	-0.74	0.56	-0.82
2	0.77	-0.47	0.71	-0.60
3	0.77	-0.47	0.76	-0.49
4	0.78	-0.48	0.78	-0.48
5	0.78	-0.48	0.79	-0.49
7	0.78	-0.47	0.80	-0.48
10	0.78	-0.47	0.81	-0.47
15	0.79	-0.47	0.81	-0.48
20	0.80	-0.47	0.82	-0.48
30	0.80	-0.47	0.82	-0.48
40	0.81	-0.47	0.83	-0.47
50	0.81	-0.47	0.83	-0.48
70	0.81	-0.46	0.83	-0.48
100	0.81	-0.47	0.83	-0.48
150	0.81	-0.47	0.82	-0.47
200	0.81	-0.47	0.83	-0.49
300	0.81	-0.48	0.83	-0.48
	**SERPINA5 (serpin family A member 5)**			
	**SomaScan to Olink**		**Olink to SomaScan**	
**K Features**	**Model Performance**	**Imputed Correlation**	**Model Performance**	**Imputed Correlation**
1	0.31	-0.06	0.58	-0.36
2	0.45	-0.41	0.60	-0.32
3	0.45	-0.42	0.71	-0.34

4	0.46	-0.43	0.71	-0.31
5	0.47	-0.43	0.75	-0.31
7	0.50	-0.43	0.76	-0.31
10	0.51	-0.40	0.76	-0.32
15	0.53	-0.39	0.76	-0.31
20	0.54	-0.37	0.77	-0.30
30	0.54	-0.38	0.78	-0.31
40	0.55	-0.38	0.79	-0.29
50	0.55	-0.37	0.79	-0.29
70	0.55	-0.38	0.79	-0.29
100	0.56	-0.40	0.79	-0.28
150	0.55	-0.40	0.79	-0.29
200	0.56	-0.41	0.80	-0.29
300	0.57	-0.42	0.80	-0.29
	**SVEP1(sushi, von Willebrand factor type A, EGF and pentraxin domain containing 1)[1]**			
	**SomaScan to Olink**		**Olink to SomaScan**	
**K Features**	**Model Performance**	**Imputed Correlation**	**Model Performance**	**Imputed Correlation**
1			0.50	
2			0.55	
3			0.57	
4			0.59	
5			0.62	
7			0.62	

10			0.66	
15			0.69	
20			0.69	
30			0.70	
40			0.71	
50			0.72	
70			0.72	
100			0.71	
150			0.72	
200			0.72	
300			0.73	

[1]Since SVEP1 is a SomaScan-only protein, it doesn't have SomaScan to Olink direction nor does it have imputed correlation.
Model performance defined as Pearson correlation between model imputed value (SomaScan-imputed Olink and measured Olink in SomaScan to Olink direction, or Olink-imputed SomaScan and measured SomaScan in Olink to SomaScan direction). Imputed correlation is defined as the Pearson correlation between SomaScan and SomaScan-imputed Olink (SomaScan to Olink direction), or between Olink and Olink-imputed SomaScan (Olink to SomaScan direction).
Ablation score calculated using first K feature based on the feature importance (e.g., K features = 5, first 5 most important features) to train in MESA train set and re-calculated model performance and imputed correlation in MESA test set.
